# Base‐Free Pd‐Catalyzed C−Cl Borylation of Fluorinated Aryl Chlorides

**DOI:** 10.1002/chem.202004648

**Published:** 2021-01-18

**Authors:** Yudha P. Budiman, Sabine Lorenzen, Zhiqiang Liu, Udo Radius, Todd B. Marder

**Affiliations:** ^1^ Institute for Inorganic Chemistry Julius-Maximilians-Universität Würzburg Am Hubland 97074 Würzburg Germany; ^2^ Institute for Sustainable Chemistry & Catalysis with Boron Julius-Maximilians-Universität Würzburg Am Hubland 97074 Würzburg Germany; ^3^ Department of Chemistry Faculty of Mathematics and Natural Sciences Universitas Padjadjaran 45363 Jatinangor Indonesia

**Keywords:** boronate ester, borylation, cross-coupling, fluoroarene, palladium-catalyzed

## Abstract

Catalytic C−X borylation of aryl halides containing two *ortho*‐fluorines has been found to be challenging, as most previous methods require stoichiometric amounts of base and the polyfluorinated aryl boronates suffer from protodeboronation, which is accelerated by *ortho*‐fluorine substituents. Herein, we report that a combination of Pd(dba)_2_ (dba=dibenzylideneacetone) with SPhos (2‐dicyclohexylphosphino‐2’,6’‐dimethoxybiphenyl) as a ligand is efficient to catalyze the C‐Cl borylation of aryl chlorides containing two *ortho*‐fluorine substituents. This method, conducted under base‐free conditions, is compatible with the resulting di*‐ortho*‐fluorinated aryl boronate products which are sensitive to base.

## Graphical abstract

**The best base is no base**! We report an efficient method for the palladium‐catalyzed C−Cl borylation of aryl chlorides containing two *ortho*‐fluorines. The reactions are conducted under base‐free conditions to avoid decomposition of the products which are sensitive to base.

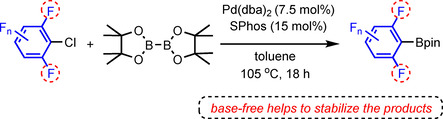

## Introduction

**Figure 1:**
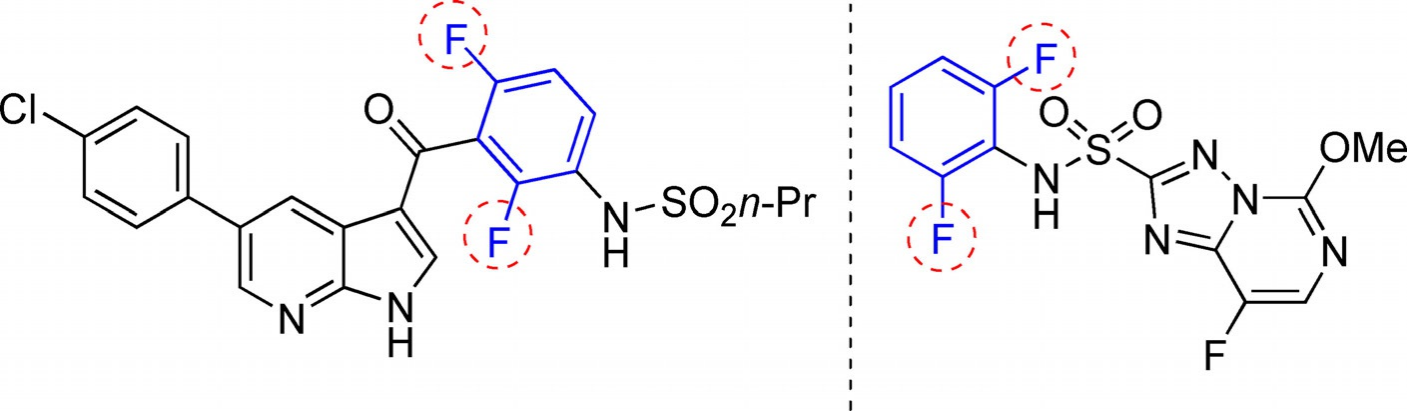
Di‐*ortho*‐fluorinated arene‐containing drugs and agrochemicals: Vemuravenib (left) used for treatment of late‐stage of melanoma. Florasulam (right) is used as a herbicide.[^1a^, ^2c^]

**Figure 2:**
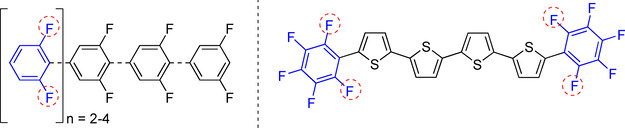
Fluoroarenes in materials science: 2,6‐difluorinated oligophenyls (left) and fluoroarene‐thiophene oligomer (right) for semiconductors.[^3c^, ^3d^]

Fluorine‐containing organic molecules have found many applications in pharmaceuticals,^[1]^ agrochemicals,^[2]^ and organic materials.^[3]^ Currently, one‐third of the top performing drugs on the market possess fluorine in their structures,[^1^, ^2^, ^3^, ^4^, ^5^, ^6^, ^7^, ^8^] and some contain two *ortho*‐fluorine substituents (Figure 1 and Figure 2). As a source of naturally occurring aryl fluorides has not been identified, this class of molecules must be produced through chemical synthesis.

Currently, one promising methodology to produce functionalized fluorine‐containing organic compounds utilizes borylation chemistry, and the resulting boryl group can be converted into diverse functional groups.^[1i]^ Recently, transition metal‐catalyzed C−H borylation of arenes with *ortho*‐to‐fluorine selectivity has been reported.[^4^, ^5^, ^6^] However, contamination by side products occurring from borylation *meta*‐ or *para*‐to‐fluorine positions led to difficulty in purification. Thus, C−X borylation reactions (X=halide) provide an option for introducing boronate ester groups selectively into fluorinated aryl halides.^[7]^

Catalytic processes to convert di‐*ortho*‐fluorinated aryl halides into boronate analogues are difficult as most such methodologies require use of stoichiometric amounts of base,^[7]^ and the resulting di‐*ortho*‐fluorinated aryl boronate derivatives are not stable under such conditions due to protodeboronation^[8]^ which is accelerated by *ortho*‐fluorine substituents. The mechanism of protodeboronation of aryl boronic acids containing two *ortho*‐fluorine substituents was examined by Lloyd‐Jones et al.^[8a]^ via experimental and computational studies. They reported that rapid protodeboronation of di‐*ortho*‐fluorinated aryl boronic acids occurs via C−B heterolysis of a trihydroxy organoboronate intermediate ([M]^+^[ArB(OH)_3_]^−^), which notably does not need water at this step. Subsequently, proton transfer from water led to the hydrolyzed product. Twenty isomers of C_6_F_5−*n*_H_n_B(OH)_2_ were studied with half‐lives (t_1/2_) spanning 9 orders of magnitude, from <3 milliseconds to 6.5 months, and it was observed that *ortho*‐fluorinated aryl boronic acids accelerated protodeboronation, with C_6_F_5_B(OH)_2_ showing the fastest rate.^[8a]^ Protodeboronation also occurred with di‐*ortho*‐fluorinated aryl‐Bpin (Bpin=4,4,5,5‐tetramethyl‐1,3,2‐dioxaborolanyl) compounds although the rate is slower than that for ‐B(OH)_2_ analogues.[^4a^, ^8e^]

**Scheme 1:**
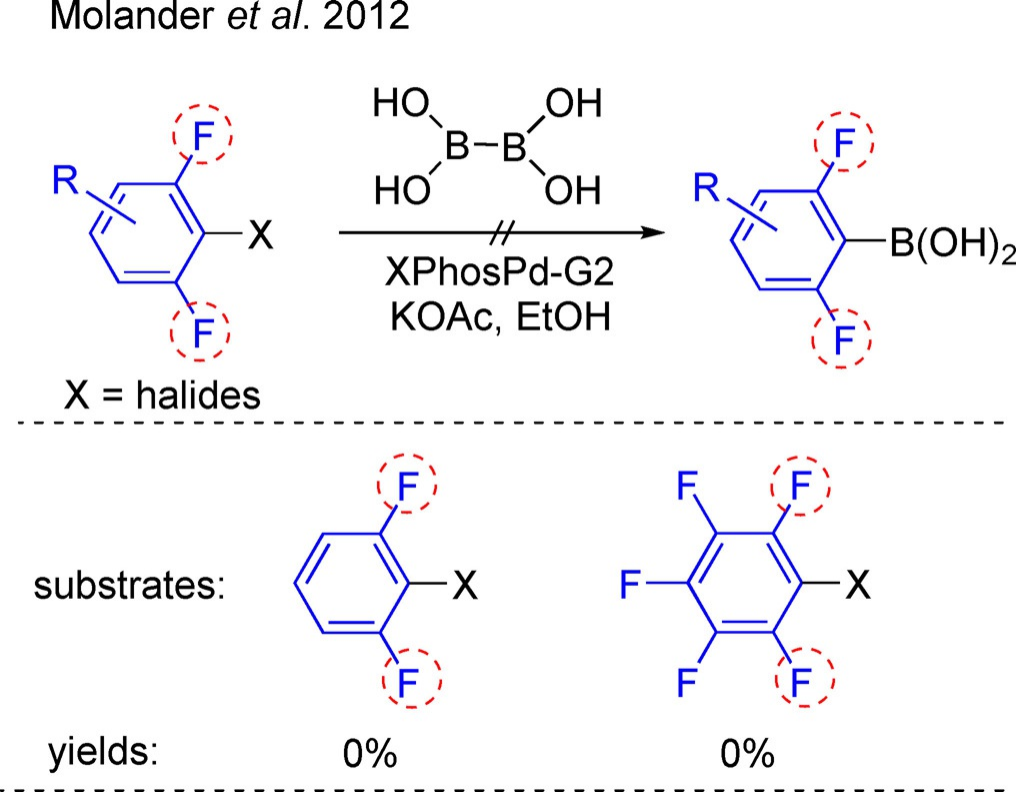
Previous efforts to borylate di‐*ortho*‐fluorinated aryl‐halides.

In 2012, Molander et al. reported borylation of aryl‐X (X=Br, Cl, I, OTf) with B_2_(OH)_4_ using the second‐generation Buchwald precatalyst XPhosPd‐G2, followed by the conversion of the aryl boronic acids into potassium trifluoroborate analogues. This method is effective to generate borylated products in good to excellent yields. However, if the C−X bond is flanked by two C−F bonds, e.g., 2‐bromo‐1,3‐difluorobenzene and 1‐chloro‐2,3,4,5,6‐pentafluorobenzene, the borylations were difficult (Scheme 1).^[7l]^ Thus, effective catalytic C−X borylation methods to convert 2,6‐di‐*ortho*‐fluorinated aryl halides into boronate analogues remain challenging.

Previously, the only reliable aryl halide C−X borylation methods to generate aryl boronates containing two *ortho*‐fluorines were stoichiometric processes via conversion of fluorinated aryl halides into aryl lithium or aryl Grignard reagents followed by addition of trialkoxyborates to generate fluorinated aryl trialkoxyborates.[^8a^, ^9^] Subsequent addition of HCl led to the generation of the corresponding boronic acid. Unfortunately, these traditional methodologies suffered from low yields and harsh reaction conditions as both aryl lithium and Grignard reagents react violently with oxygen and moisture and decompose readily, and the formation of stoichiometric metal salts made isolation of the desired products difficult.

Recently, we have reported thermal^[10a]^ and photocatalytic^[10b]^ C−F borylation of partially fluorinated arenes using NHC nickel complexes (NHC=N‐Heterocyclic Carbene) to generate fluorinated aryl‐Bpin compounds in fair to excellent yields. The reactions are effective and selective to give fluorinated aryl boronates containing one or no *ortho*‐fluorines, but not selective to generate aryl boronates containing two *ortho*‐fluorines. Notably, the borylation of C_6_F_6_ to generate C_6_F_5_Bpin gave a low yield.

Very recently, we have reported applications of fluorinated aryl boronate substrates, including those containing two *ortho*‐fluorines, in Suzuki–Miyaura cross‐couplings^[11a]^ and oxidative cross‐couplings with terminal alkynes^[11b]^ using copper catalysts, and homocoupling reactions using palladium catalysts.^[12]^ Compared to aryl bromides and iodides, aryl chlorides are less expensive and more readily available. Herein we report a method to provide di‐*ortho*‐fluorinated aryl boronates, via the palladium‐catalyzed C−Cl borylation of di‐*ortho*‐fluorinated aryl chlorides using B_2_pin_2_ in good to excellent yields. This method is conducted under base‐free conditions, thus, stabilizing the di‐*ortho*‐fluorinated boronate products from decomposition.

## Results and Discussion

**Scheme 2:**
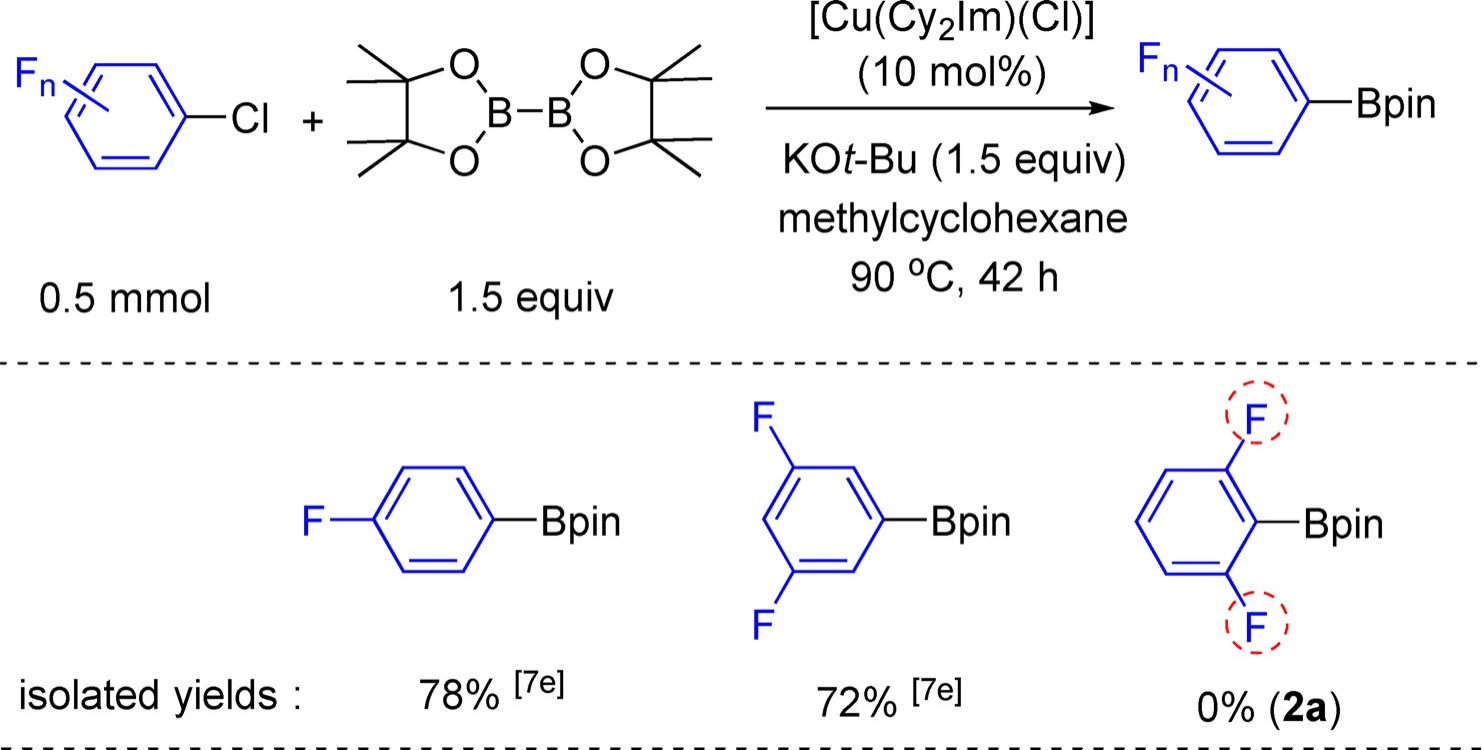
Cu‐catalyzed C–Cl borylation of fluorinated aryl chlorides.

We previously reported that copper NHC complexes are efficient catalysts for C−Cl borylation in the presence of stoichiometric amounts of a strong base such as potassium *tert*‐butoxide (KO*t*Bu), in methylcyclohexane, at 90 °C. These conditions were efficient to generate fluorinated aryl‐Bpin compounds such as 4‐fluorophenyl‐Bpin, and 3,5‐difluorophenyl‐Bpin, in which the fluorines are present in *para*‐ or di‐*meta*‐positions (Scheme 2).^[7f]^ We tested this set of conditions employing the challenging substrate 2‐chloro‐1,3‐difluorobenzene, in which fluorines are present in both *ortho*‐positions. However, this method failed as no borylated products were detected (Scheme 2). It should be noted that Frohn and Adonin et al. examined the treatment of fluorinated aryl boronic acids with wet pyridine.^[8c]^ Unlike *ortho*‐fluorinated aryl boronic acid derivatives which are protodeboronated rapidly, interestingly, electron‐deficient aryl boronic acids with no *ortho*‐fluorines, such as 3,4,5‐trifluorophenyl boronic acid, are stable towards protodeboronation even at 100 °C after 3 h.

**Scheme 3:**
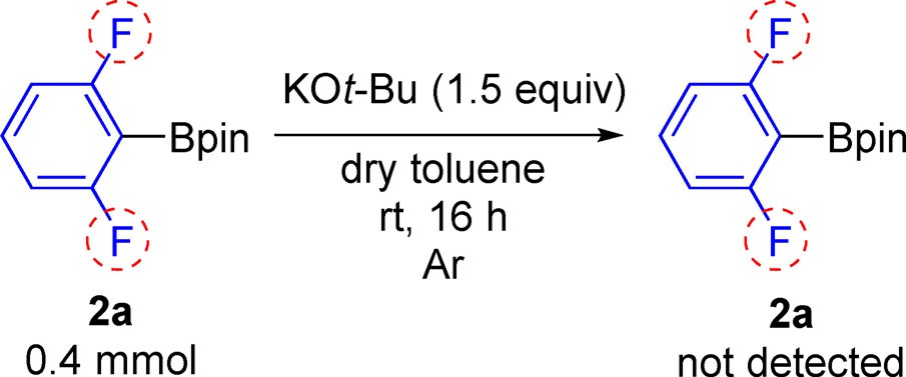
KO*t*Bu‐promoted decomposition of 2,6‐di‐fluorophenyl‐Bpin.

We believed that such a low yield of **2 a** (Scheme 2) is due to the decomposition of the borylated product 2,6‐fluorophenyl‐Bpin in the presence of the strong base KO*t*Bu. Thus, we reacted 0.4 mmol of 2,6‐fluorophenyl‐Bpin, with 0.6 mmol of KO*t*Bu in a vial containing 4 mL of dry toluene with stirring at room temperature for 16 h under argon (Scheme 3). Afterward, the reaction mixture was examined by ^19^F{^1^H} NMR spectroscopy (under argon) and GC‐MS (in air). We found that 2,6‐difluorophenyl‐Bpin was no longer present. In addition, in 2012, Hartwig et al. reported that 2,6‐difluorophenyl‐Bpin decomposed within 30 min in a THF solution containing a weak base such as 2 m aqueous Na_2_CO_3_, at 50 °C.^[4a]^ In 2018, Carrow et al. treated C_6_F_5_Bpin and C_6_F_5_B(OH)_2_ with wet triethylamine (weak base), and found that C_6_F_5_Bpin underwent protodeboronation, but the rate was slower than that for C_6_F_5_B(OH)_2_.^[8e]^ Hence, it can be suggested that the best conditions to synthesize 2,6‐difluorophenyl‐Bpin derivatives are base‐free methods. Previously, we tried to use our optimized conditions with the NHC‐copper complex to catalyze the C−Cl borylation of aryl chlorides, without base, but it did not work.^[7f]^

**Figure 3:**
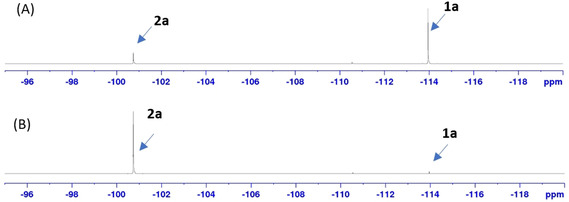
^19^F{^1^H} NMR spectra of reaction mixtures: (A) conditions stated in Table 1, entry 1; (B) conditions stated in Table 1, entry 2.

Recently, Matsubara and Yorimitsu et al. reported that a combination of catalytic amounts of Pd_2_(dba)_3_ (tris(dibenzylideneacetone)dipalladium(0)) and SPhos (2‐dicyclohexylphosphino‐2’,6’‐dimethoxybiphenyl) in toluene under base‐free conditions, is effective to catalyze C−Cl borylation of aryl chlorides, using B_2_pin_2_ as the boron source. However, aryl chloride substrates containing two *ortho*‐fluorine substituents were not examined.^[13]^ Hence, we used the Matsubara and Yorimitsu conditions, with 1,5‐difluoro‐6‐chloro‐benzene, with stirring for 18 h at 105 °C. Surprisingly, it is not efficient as only a 17 % yield of product was isolated (Table 1, entry 1). Interestingly, using Pd(dba)_2_ instead of Pd_2_(dba)_3_ increased the efficiency to 92 % yield. We repeated the reactions under the conditions in Table 1, entries 1 and 2, and in situ ^19^F{^1^H} NMR spectroscopy confirmed that a combination Pd(dba)_2_ as a precatalyst and SPhos as a ligand gave 99 % conversion (Figure 3). We also utilized both commercial and freshly prepared samples of Pd_2_(dba)_3_ with similar results. The lower activity of Pd_2_(dba)_3_ compared with that of Pd(dba)_2_ is likely a result of the lower solubility of the former in toluene. Using the Pd^II^ salt Pd(OAc)_2_ proved inefficient (Table 1, entry 3). Other ligands such as P*t*Bu_3_ (tri‐*tert*‐butylphosphine) and JohnPhos (2‐(di‐*tert*‐butylphosphino)biphenyl) in combination with Pd(dba)_2_ decreased the efficiency (Table 1, entries 4 and 5). No reactions were observed using bidentate phosphine or nitrogen‐based ligands such as XantPhos (4,5‐bis(diphenylphosphino)‐9,9‐dimethylxanthene), DPPP (1,3‐bis(diphenylphosphino)propane), or phenanthroline (Table 1, entries 6–8).

**Table 1:** Reaction conditions screened for the Pd‐catalyzed borylation of 1,5‐difluoro‐6‐chloro‐benzene.

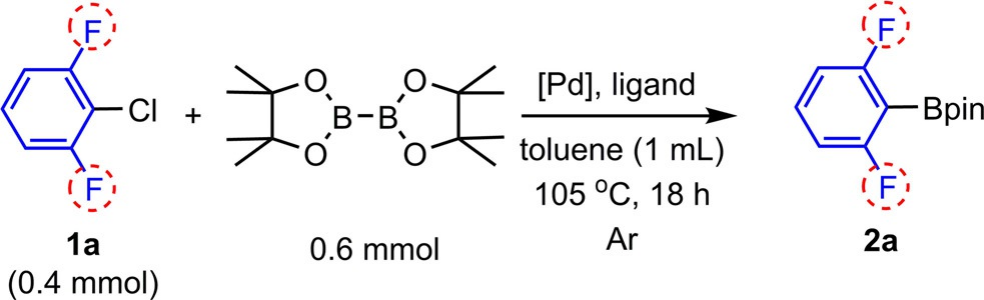			
Entry	[Pd]	Ligand	Isolated yield
1	Pd_2_(dba)_3_ (4 mol %)	SPhos (16 mol %)	17 %
**2**	**Pd(dba)_2_ (7.5 mol %)**	**SPhos (15 mol %)**	**92 %**
3	Pd(OAc)_2_ (8 mol %)	SPhos (16 mol %)	11 %
4	Pd(dba)_2_ (8 mol %)	P*t*Bu_3_ (16 mol %)	61 %
5	Pd(dba)_2_ (8 mol %)	JohnPhos (16 mol %)	35 %
6	Pd(dba)_2_ (8 mol %)	XantPhos (8 mol %)	0 %
7	Pd(dba)_2_ (8 mol %)	DPPP (8 mol %)	0 %
8	Pd(dba)_2_ (8 mol %)	phenanthroline (8 mol %)	0 %

**Scheme 4:**
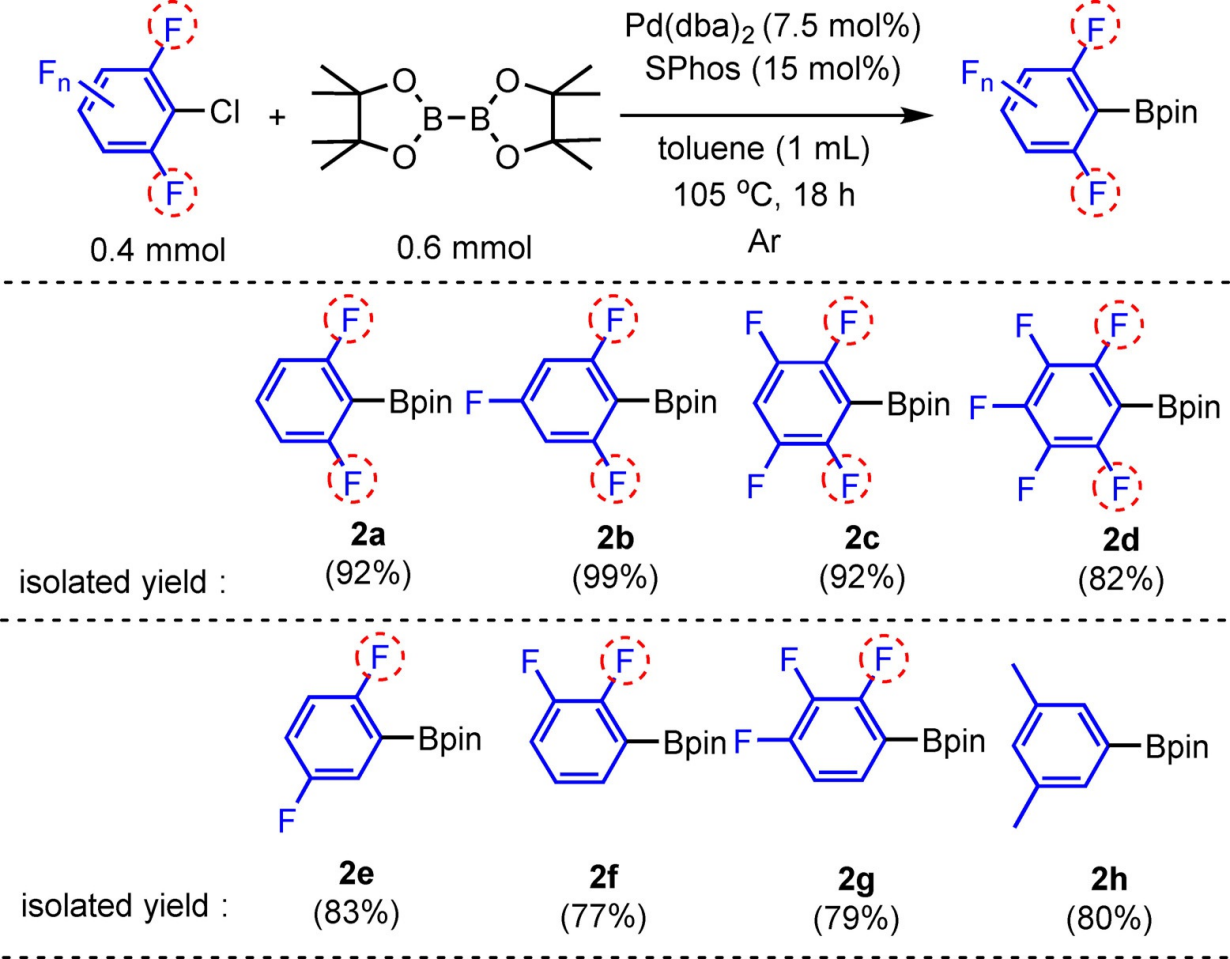
Reaction scope for the borylation of fluorinated aryl chloride derivatives.

Having optimal conditions in hand, we expanded the scope reactions for other fluorinated aryl chloride substrates (Scheme 4), thus generating di‐*ortho*‐fluorinated aryl‐Bpin derivatives (**2 a**–**d**) via catalytic C−Cl borylation in excellent yields. The substrate scope was expanded to include aryl chlorides containing one or no *ortho*‐fluorines and the products (**2 e**–**h**) were isolated in good to very good yields.

**Scheme 5:**
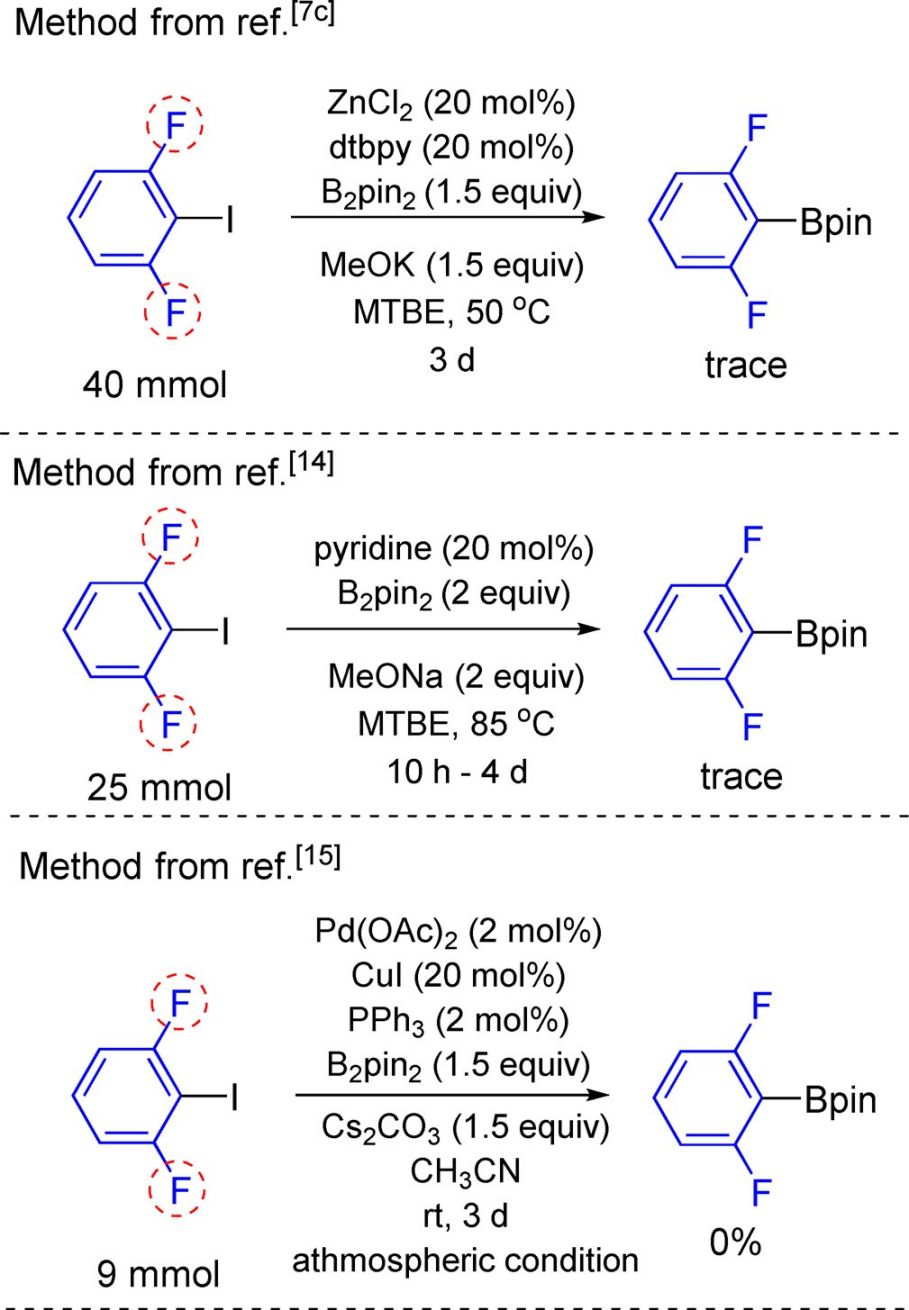
Attempted borylation of di‐*ortho*‐fluorinated aryl halides using established methods that require stoichiometric amounts of base.

We also attempted to use other established methods for the C−X borylation of aryl halides containing two *ortho*‐fluorine substituents. Notably, those methods require stoiciometric amounts of base, and proved efficient to generate aryl boronates in good to excellent yields.[^7c^, ^14^, ^15^] However, employing aryl halides containing two *ortho*‐fluorines were not examined in the previous studies. Thus, we attempted to use those methods to employ aryl halides containing two *ortho*‐fluorine substituents. Not surprisingly, they proved inefficient (Scheme 5).

## Conclusions

In summary, we report a base‐free catalytic method for C−Cl borylation of aryl chlorides containing two *ortho*‐fluorines using a combination of Pd(dba)_2_ and SPhos as a ligand, to generate previously challenging di‐*ortho*‐fluorinated aryl‐Bpin products. Our base‐free conditions prevent the decomposition of the di‐*ortho*‐fluorinated aryl boronate products. This method provides a nice alternative to traditional approaches using lithium or Grignard reagents.[^8a^, ^9^] The fluorinated aryl boronate products are useful substrates for Suzuki–Miyaura cross‐coupling with aryl halides,^[11a]^ oxidative cross‐coupling with terminal alkynes,^[11b]^ homocoupling reactions,^[12]^ etc.^[1i]^

## Experimental Section

**General procedure for Pd‐catalyzed C−Cl borylation**: In an argon‐filled glove box, to a dried vial equipped with a stirring bar, and containing 1 mL of toluene, were added Pd(dba)_2_ (17 mg, 0.03 mmol), and SPhos (25 mg, 0.06 mmol), and the mixture was stirred until homogeneous. Then, B_2_pin_2_ (152 mg, 0.6 mmol) and the corresponding fluorinated aryl chlorides (0.4 mmol) were added. After sealing the vial and removing it from the glovebox, the suspension was stirred for 18 h at 105 °C. After cooling to room temperature, the solvent was evaporated in vacuo and the residue was purified by flash column chromatography on silica gel (ethyl acetate : hexane=2:98) to obtain the corresponding product, which was crystallized in a freezer (−30 °C).

## Conflict of interest

The authors declare no conflict of interest.

## Supporting information

As a service to our authors and readers, this journal provides supporting information supplied by the authors. Such materials are peer reviewed and may be re‐organized for online delivery, but are not copy‐edited or typeset. Technical support issues arising from supporting information (other than missing files) should be addressed to the authors.

Supplementary
