# Supplementary material for: Base‐Free Pd‐Catalyzed C−Cl Borylation of Fluorinated Aryl Chlorides
Source: Chemistry. 2021 Jan 18;27(11):3869–74. doi: 10.1002/chem.202004648 (PMC7986610; doi:10.1002/chem.202004648)
Supplement: Supplementary file 1 — Supplementary [file CHEM-27-3869-s001.pdf]

Chemistry–A European Journal

Supporting Information

Base-Free Pd-Catalyzed C−Cl Borylation of Fluorinated Aryl Chlorides

Yudha P. Budiman,[a, b, c] Sabine Lorenzen,[a, b] Zhiqiang Liu,[a, b] Udo Radius,*[a] and Todd B. Marder*[a, b]

Table of Contents

1. General Information S2

2. General Procedure for Pd-Catalyzed C–Cl Borylation S3

3. Characterization (GC-MS, NMR and HRMS data) of Products S3

4. ^{1}H, $^{13}C\{^{1}H\}$, ^{19}F, $^{19}F\{^{1}H\}$, $^{11}B\{^{1}H\}$ NMR Spectra and GC-MS Data S8

References S32

1. General Information

Unless otherwise noted, all reagents were purchased from Alfa-Aesar, Sigma-Aldrich, Fluorochem, Apollo Chemicals, Acros, OxChem and were checked for purity by GC-MS and/or ^{1}H NMR spectroscopy and used as received. B_2pin_2 was kindly provided by AllyChem Co. Ltd. (Dalian, China). HPLC grade solvents were argon saturated, dried using an Innovative Technology Inc. Pure-Solv Solvent Purification System, and further deoxygenated using the freeze-pump-thaw method.

Automated flash chromatography was performed using silica gel (Biotage SNAP cartridge KP-Sil 10 g and KP-Sil 25 g) using a Biotage® Isolera Four system. Commercially available, precoated TLC plates (Polygram® Sil G/UV254) were purchased from Machery-Nagel. The removal of solvent was performed on a rotary evaporator *in vacuo* at a maximum temperature of 40 °C.

GC-MS analyses were performed using an Agilent 7890A gas chromatograph (column: HP-5MS 5% phenylmethylsiloxane, 10 m, Ø 0.25 mm, film 0.25 μm; injector: 250 °C; oven: 40 °C (2 min), 40 °C to 280 °C (20 °C·min^{-1}); carrier gas: He (1.2 mL min^{-1}) equipped with an Agilent 5975C inert MSD with triple-axis detector operating in EI mode and an Agilent 7693A series auto sampler/injector. HRMS analyses were performed using a Thermo Fischer Scientific Exactive Plus Orbitrap MS system (APCI and ASAP probe).

All NMR spectra were recorded at 298 K using Bruker Avance I 500 (^{1}H NMR, 500 MHz; ^{13}C{^{1}H} NMR, 126 MHz; ^{19}F NMR, 471 MHz), Bruker DRX-300 (^{13}C{^{1}H} 75 MHz; ^{11}B{^{1}H}, 96 MHz), or Bruker Avance Neo (^{1}H NMR, 400 MHz; ^{13}C{^{1}H} NMR, 101 MHz; ^{19}F{^{1}H} NMR, 377 MHz) spectrometers. ^{1}H NMR chemical shifts are reported relative to TMS and were referenced via residual proton resonances of the corresponding deuterated solvent ($CDCl_3$: 7.26 ppm), ^{13}C{^{1}H} NMR spectra are reported relative to TMS via the carbon signals of the deuterated solvent ($CDCl_3$: 77.16 ppm), ^{19}F NMR and ^{19}F{^{1}H} spectra are reported

relative to external $CFCl_3$, and $^{11}B\{^1H\}$ NMR chemical shifts are quoted relative to $BF_3{\cdot}Et_2O$ as external standard. In the $^{19}F\{^1H\}$ and ^{19}F NMR spectra of some of the products containing fluorine atoms *ortho* to the boryl group, a smaller set of signals can be observed slightly downfield from the larger signals, with an integration ratio of ca. 20:80, which is the result of the natural abundance of the ^{10}B and ^{11}B isotopes. As the isotope effect is relatively small, it is only observed for most downfield shifted resonances assigned to the ortho fluorines, as these F atoms are closest to the boron atom.

2. General Procedure for Pd-Catalyzed C–Cl Borylation

In a glove box, under an argon atmosphere, into a dried vial equipped with a stirring bar and containing 1 mL of toluene, were added $Pd(dba)_2$ (17 mg, 0.03 mmol), and SPhos (25 mg, 0.06 mmol), and the mixture was stirred until homogeneous. Then, B_2pin_2 (152 mg, 0.6 mmol) and the corresponding fluorinated aryl chlorides (0.4 mmol) were added. After sealing the vial and removal from the glove box, the suspension was stirred for 18 h at 105 ˚C. After cooling to room temperature, the solvent was evaporated *in vacuo* and the residue was purified by flash column chromatography on silica gel (ethyl acetate : hexane = 2 : 98) and the product was crystallized in a freezer (-30 °C).

3. Characterization (GC-MS, NMR and HRMS data) of Products

2-(2,6-difluorophenyl)-4,4,5,5-tetramethyl-1,3,2-dioxaborolane (2a)

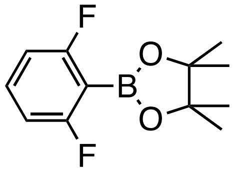

Isolated yield: 92% (88 mg, pale yellow solid). ^1H NMR (400 MHz, $CDCl_3$) δ = 7.35 (*tt*, *J* = 8, 7 Hz, 1H), 6.87–6.81 (*m*, 2H), 1.38 (*s*, 12H); $^{19}C\{^1H\}$ NMR (100 MHz, $CDCl_3$) δ = 166.8 (*dd*, $^1J_{C\text{-}F}$ = 250 Hz, $^3J_{C\text{-}F}$ = 13 Hz), 133.2 (*t*, $^3J_{C\text{-}F}$ = 11 Hz), 111.2 (*m*), 84.4, 24.9; $^{19}F\{^1H\}$ NMR (377 MHz, $CDCl_3$) δ = 100.7 (*s*, 2F); $^{11}B\{^1H\}$ NMR (160 MHz, $CDCl_3$) δ = 29.8; GC-

MS: [t = 9.196 min] m/z: 240 $[M]^+$; HRMS (APCI) calcld for $[C_{12}H_{16}BF_2O_2^+]$ $[M+H]^+$: 241.1206; found 241.1195. Spectroscopic data matched those in the literature.[S1]

4,4,5,5-tetramethyl-2-(2,4,6-trifluorophenyl)-1,3,2-dioxaborolane (2b)

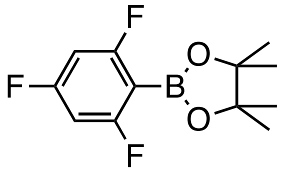

Isolated yield: 99% (102 mg, pale yellow solid). ^{1}H NMR (500 MHz, $CDCl_3$) δ = 6.62–6.57 (*m*, 2H), 1.36 (*s*, 12H); ^{19}C{^{1}H} NMR (126 MHz, $CDCl_3$) δ = 167.4 (*dm*, $^1J_{C\text{-}F}$ = 252 Hz), 165 (*dt*, $^1J_{C\text{-}F}$ = 252 Hz, $^3J_{C\text{-}F}$ = 16 Hz), 100.2 (*m*), 84.4, 24.8; ^{19}F{^{1}H} NMR (377 MHz, $CDCl_3$) δ = -97.2 (*d*, $^3J_{F\text{-}F}$ = 9 Hz, 2F), -103.1 (*t*, $^3J_{F\text{-}F}$ = 9 Hz, 1F); ^{11}B{^{1}H} NMR (128 MHz, $CDCl_3$) δ = 29.4; GC-MS: [t = 8.763 min] m/z: 258 $[M]^+$; HRMS (ASAP) Calcld for $[C_{12}H_{15}BO_2F_3]^+$ $[M+H]^+$: 259.1112; found 259.1104. Spectroscopic data matched those in the literature.[S1]

4,4,5,5-tetramethyl-2-(2,3,5,6-tetrafluorophenyl)-1,3,2-dioxaborolane (2c)

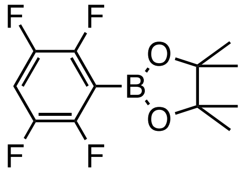

Isolated yield: 92% (101 mg, pale yellow solid). ^{1}H NMR (400 MHz, $CDCl_3$) δ = 7.10 (*tt*, $^3J_{H\text{-}F}$ = 9 Hz, $^4J_{H\text{-}F}$ = 7 Hz, 1H), 1.38 (*s*, 12H); ^{19}C{^{1}H} NMR (101 MHz, $CDCl_3$) δ = 148.8 (*dm*, $^1J_{C\text{-}F}$ = 251 Hz), 144.6 (*dm*, $^1J_{C\text{-}F}$ = 253 Hz), 108.7 (*tt*, $^2J_{C\text{-}F}$ = 22 Hz, $^3J_{C\text{-}F}$ = 2 Hz), 85.1, 24.8; ^{19}F{^{1}H} NMR (377 MHz, $CDCl_3$) δ = -131.0 (*dd*, $J_{F\text{-}F}$ = 22 Hz, $J_{F\text{-}F}$ = 15 Hz), -139.3 (*dd*, $J_{F\text{-}F}$ = 22 Hz, $J_{F\text{-}F}$ = 15 Hz, 2F); ^{11}B{^{1}H} NMR (128 MHz, $CDCl_3$) δ = 29.3 Hz; GC-MS: [t = 8.039 min] m/z: 276 $[M]^+$; HRMS (ASAP) Calcld for $[C_{12}H_{14}BO_2F_4]^+$ $[M+H]^+$: 277.1017; found 277.1006. Spectroscopic data matched those in the literature.[S1]

4,4,5,5-tetramethyl-2-(perfluorophenyl)-1,3,2-dioxaborolane (2d)

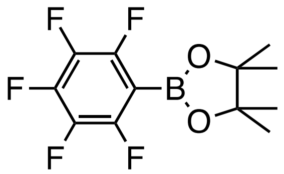

Isolated yield: 82% (96 mg, pale yellow solid). ^{1}H NMR (500 MHz, $CDCl_3$) δ = 1.38 (*s*, 12H); ^{19}C{^{1}H} NMR (126 MHz, $CDCl_3$) δ = 149.4 (*dm*, $^{1}J_{C\text{-}F}$ = 251 Hz), 143.1 (*dm*, $^{1}J_{C\text{-}F}$ = 251 Hz), 137.4 (*dm*, $^{1}J_{C\text{-}F}$ = 252 Hz), 85.2, 24.8; ^{19}F NMR (471 MHz, $CDCl_3$) δ = -129.6 (*m*, 2F), -149.8 (*m*, 1F), -261.0 (*m*, 2F); ^{11}B{^{1}H} NMR (96 MHz, $CDCl_3$) δ = 29.0; GC-MS: [t = 8.668 min] m/z: 294 [*M*]$^{+}$; HRMS (ASAP) calcd. for $[C_{12}H_{13}BF_5O_2]^+$: 295.0923 [*M*+H]$^{+}$; found: 295.0912. Spectroscopic data matched those in the literature.[S1]

2-(2,5-difluorophenyl)-4,4,5,5-tetramethyl-1,3,2-dioxaborolane (2e)

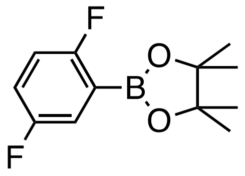

Isolated yield: 83% (80 mg, pale yellow solid) ^{1}H NMR (500 MHz, $CDCl_3$) δ = 7.40–7.36 (*m*, 1H), 7.10–7.06 (*m*, 1H), 6.99–6.95 (*m*, 1H), 1.35 (*s*, 12H); ^{13}C{^{1}H} (126 MHz, $CDCl_3$) δ = 163.1 (*dd*, $^{1}J_{C\text{-}F}$ = 247 Hz, $^{4}J_{C\text{-}F}$ = 2 Hz), 158.5 (*dd*, $^{1}J_{C\text{-}F}$ = 242 Hz, $^{4}J_{C\text{-}F}$ = 2 Hz), 122.4 (*dd*, $^{2}J_{C\text{-}F}$ = 22 Hz, $^{3}J_{C\text{-}F}$ = 9 Hz), 119.8 (*dd*, $^{2}J_{C\text{-}F}$ = 24 Hz, $^{3}J_{C\text{-}F}$ = 9 Hz), 116.7 (*dd*, $^{2}J_{C\text{-}F}$ = 27 Hz, $^{3}J_{C\text{-}F}$ = 8 Hz), 84.3, 24.9; ^{19}F{^{1}H} (377 MHz, $CDCl_3$) δ = -109.4 (*d*, $^{5}J_{F\text{-}F}$ = 19 Hz, 1F), -120.6 (*d*, $^{5}J_{F\text{-}F}$ = 19 Hz, 1F); ^{11}B{^{1}H} NMR (160 MHz, $CDCl_3$) δ = 30.2; GC-MS: [t = 8.248 min] m/z: 240 [*M*]$^{+}$; HRMS (ASAP) Calcld for $[C_{12}H_{16}BO_2F_2]^+$ [*M*+H]$^{+}$: 241.1206; found 241.1201. Spectroscopic data matched those in the literature.[S2]

2-(2,3-difluorophenyl)-4,4,5,5-tetramethyl-1,3,2-dioxaborolane (2f)

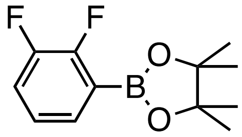

Isolated yield: 77% (74 mg, pale yellow solid). ^{1}H NMR (500 MHz, $CDCl_3$) δ = 7.47–7.44 (*m*, 1H), 7.26–7.21 (*m*, 1H), 7.09–7.05 (*m*, 1H), 1.36 (*s*, 12H); ^{13}C{^{1}H} (126 MHz, $CDCl_3$) δ = 154.7 (*dd*, $^{1}J_{C\text{-}F}$ = 253 Hz, $^{2}J_{C\text{-}F}$ = 12 Hz), 150.6 (*dd*, $^{1}J_{C\text{-}F}$ = 248 Hz, $^{2}J_{C\text{-}F}$ = 14 Hz), 131.2 (*dd*, $^{3}J_{C\text{-}F}$ = 7 Hz, $^{4}J_{C\text{-}F}$ = 4 Hz), 124.2 (*dd*, $^{3}J_{C\text{-}F}$ = 6 Hz, $^{4}J_{C\text{-}F}$ = 4 Hz), 120.3 (*dd*, $^{2}J_{C\text{-}F}$ = 17 Hz, $^{3}J_{C\text{-}F}$ = 1 Hz), 84.3, 25.0; ^{19}F{^{1}H} (377 MHz, $CDCl_3$) δ = -129.0 (*d*, $^{3}J_{F\text{-}F}$ = 22 Hz, 1F), -139.1 (*d*, $^{3}J_{F\text{-}F}$ = 22 Hz, 1F); ^{11}B{^{1}H} NMR (160 MHz, $CDCl_3$) δ = 30.0 ; GC-MS: [t = 9.324 min] m/z: 240 [*M*]$^{+}$; HRMS (ASAP) Calcld for $[C_{12}H_{16}BO_2F_2]^+$ [*M*+H]$^{+}$: 241.1206; found 241.1198. Spectroscopic data matched those in the literature.[S3]

4,4,5,5-tetramethyl-2-(2,3,4-trifluorophenyl)-1,3,2-dioxaborolane (2g)

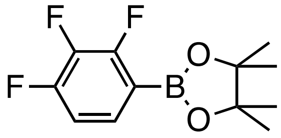

Isolated yield: 79% (82 mg, pale yellow solid). ^{1}H NMR (500 MHz, $CDCl_3$) δ = 7.45–7.40 (*m*, 1H), 6.96–6.91 (*m*, 1H), 1.34 (*s*, 12H); ^{13}C{^{1}H} (126 MHz, $CDCl_3$) δ = 155.8 (*ddd*, $^{1}J_{C\text{-}F}$ = 255 Hz, $^{2}J_{C\text{-}F}$ = 9 Hz, $^{3}J_{C\text{-}F}$ = 4 Hz), 153.6 (*ddd*, $^{1}J_{C\text{-}F}$ = 254 Hz, $^{2}J_{C\text{-}F}$ = 10 Hz, $^{3}J_{C\text{-}F}$ = 4 Hz), 140.0 ($^{1}J_{C\text{-}F}$ = 252 Hz, $^{2}J_{C\text{-}F}$ = 17 Hz, $^{2}J_{C\text{-}F}$ = 15 Hz), 130.5 (*m*), 112.4 (*dd*, $^{2}J_{C\text{-}F}$ = 17 Hz, $^{3}J_{C\text{-}F}$ = 4 Hz), 84.4, 24.9; ^{19}F{^{1}H} (377 MHz, $CDCl_3$) δ = -124.8 (*dd*, $^{3}J_{F\text{-}F}$ = 20 Hz, $^{4}J_{F\text{-}F}$ = 11 Hz, 1F), -130.1 (*dd*, $^{3}J_{F\text{-}F}$ = 20 Hz, $^{4}J_{F\text{-}F}$ = 11 Hz, 1F), -162,1 (*t*, $^{3}J_{F\text{-}F}$ = 20 Hz); ^{11}B{^{1}H} NMR (128 MHz, $CDCl_3$) δ = 29.8; GC-MS: [t = 8.249 min] m/z: 258 [*M*]$^{+}$; HRMS (ASAP) Calcld for $[C_{12}H_{15}BO_2F_3]^+$ [*M*+H]$^{+}$: 259.1112; found 259.1106. Spectroscopic data matched those in the literature.[S4]

2-(3,5-dimethylphenyl)-4,4,5,5-tetramethyl-1,3,2-dioxaborolane (2h)

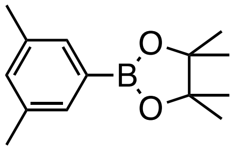

Isolated yield: 80 % (74 mg, yellow pale solid). ^{1}H NMR (500 MHz, $CDCl_3$) δ = 7.46–7.45 (*m*, 2H), 7.12–7.11 (*m*, 1H); ^{13}C{^{1}H} (75 MHz, $CDCl_3$) δ = 137.3, 133.1, 132.5, 83.8, 25.0, 21.3; ^{11}B{^{1}H} NMR (96 MHz, $CDCl_3$) δ = 30.9; GC-MS: [t = 10.374 min] m/z: 232 $[M]^+$; HRMS (APCI) Calcld for $[C_{14}H_{22}BO_2]^+$ $[M+H]^+$: 233.1707 found 233.1703. Spectroscopic data matched those in the literature.[S5]

4. ^{1}H, $^{13}C\{^{1}H\}$, ^{19}F, $^{19}F\{^{1}H\}$, $^{11}B\{^{1}H\}$ NMR Spectra and GC-MS Data

^{1}H NMR spectrum of **2a** ($CDCl_3$, 500 MHz

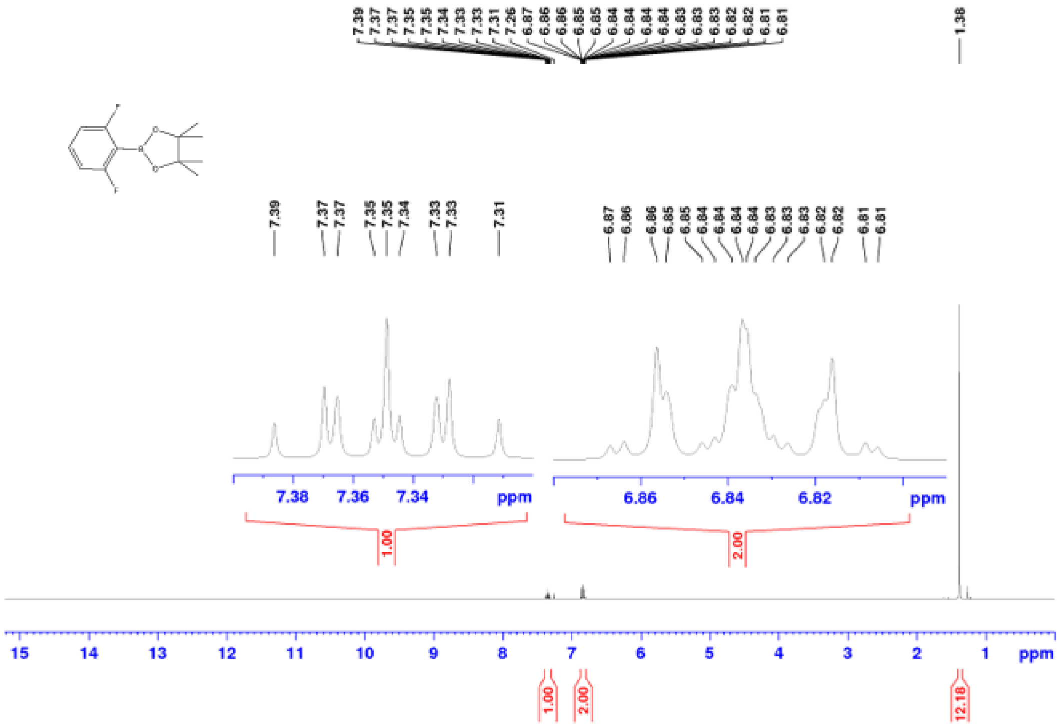

$^{13}C\{^{1}H\}$ NMR spectrum of **2a** ($CDCl_3$, 100 MHz)

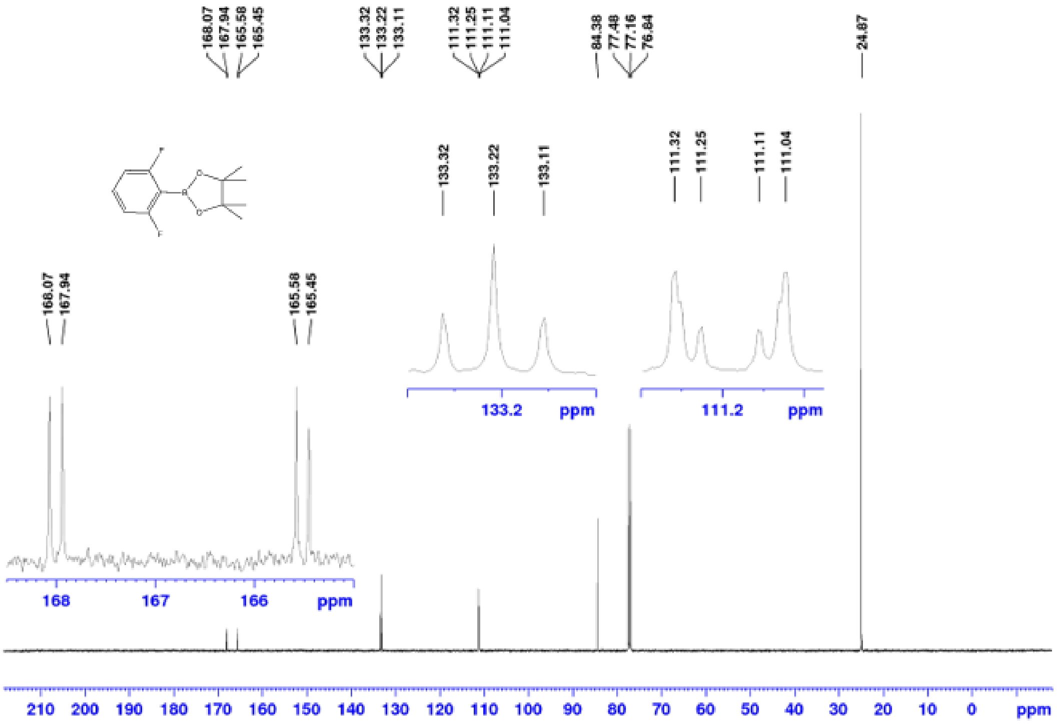

$^{19}F\{^{1}H\}$ NMR spectrum of **2a** ($CDCl_3$, 377 MHz)

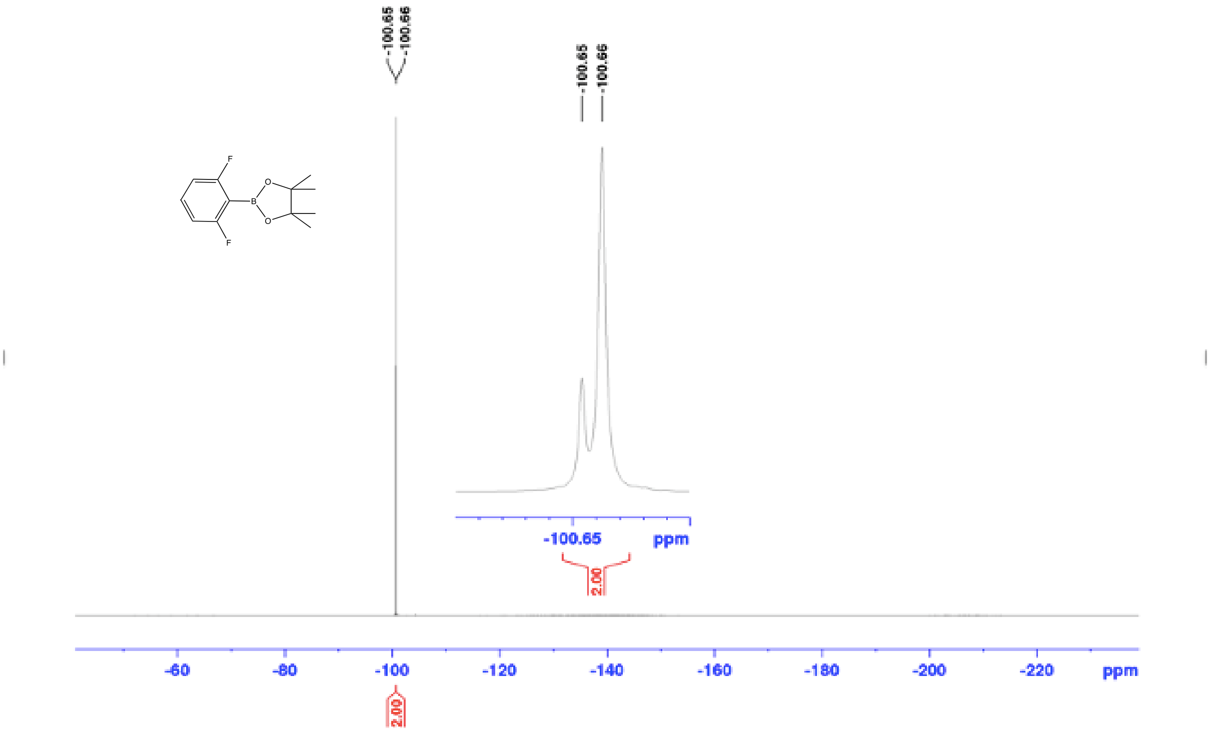

$^{11}B\{^{1}H\}$ NMR spectrum of **2a** (160 MHz, $CDCl_3$)

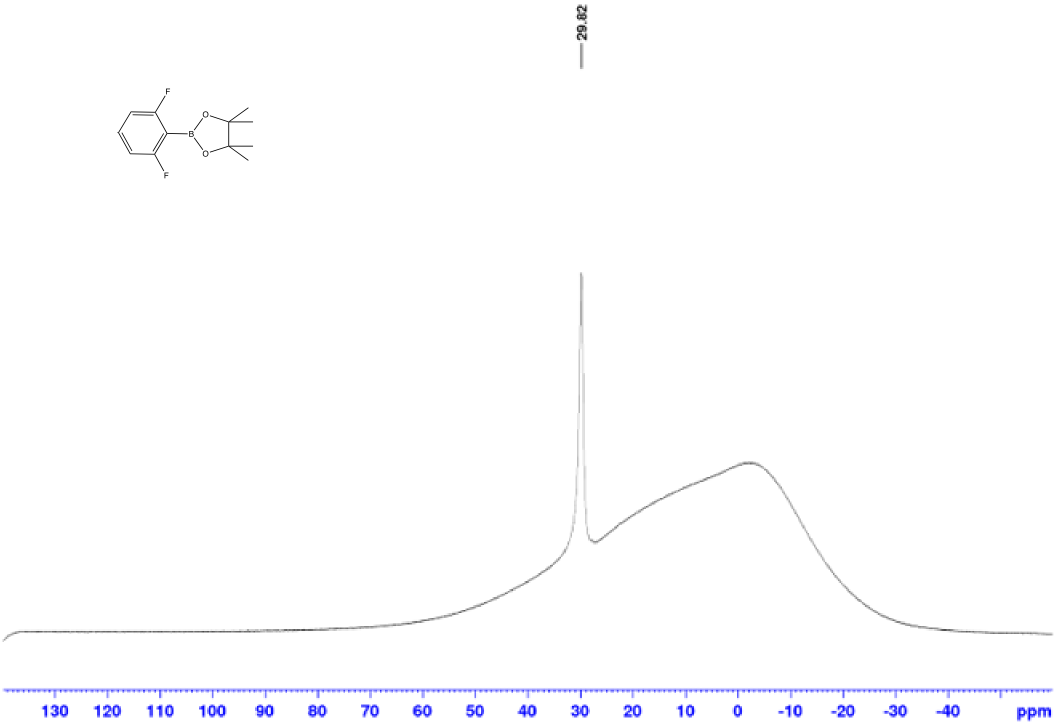

GC-MS of **2a**

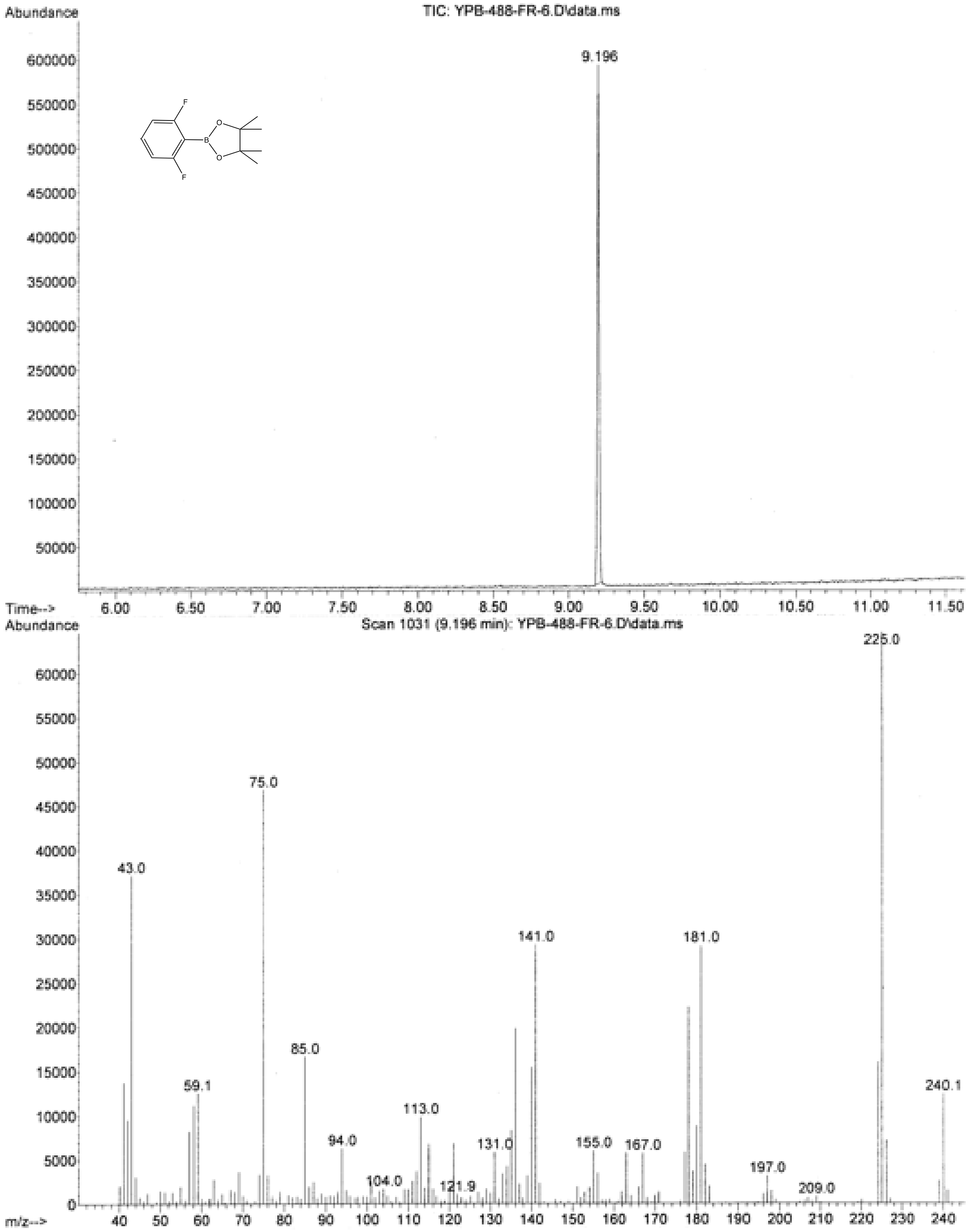
Abundance
TIC: YPB-488-FR-6.D\data.ms
9.196
600000
550000
500000
450000
400000
350000
300000
250000
200000
150000
100000
50000
Time-->
6.00 6.50 7.00 7.50 8.00 8.50 9.00 9.50 10.00 10.50 11.00 11.50
Abundance
Scan 1031 (9.196 min): YPB-488-FR-6.D\data.ms
225.0
75.0
43.0
141.0
181.0
85.0
59.1
240.1
113.0
94.0
131.0
155.0
167.0
197.0
104.0
121.9
209.0
60000
55000
50000
45000
40000
35000
30000
25000
20000
15000
10000
5000
0
m/z-->
40 50 60 70 80 90 100 110 120 130 140 150 160 170 180 190 200 210 220 230 240

^{1}H NMR spectrum of **2b** ($CDCl_3$, 500 MHz)

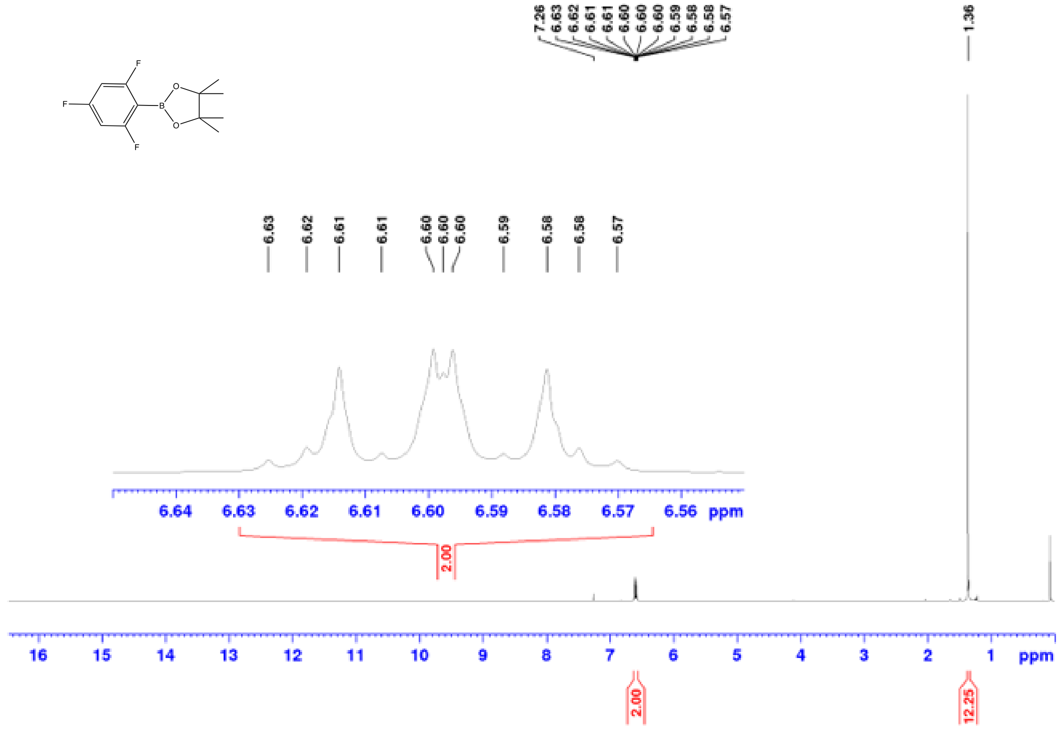

$^{13}C\{^{1}H\}$ NMR spectrum of **2b** ($CDCl_3$, 126 MHz)

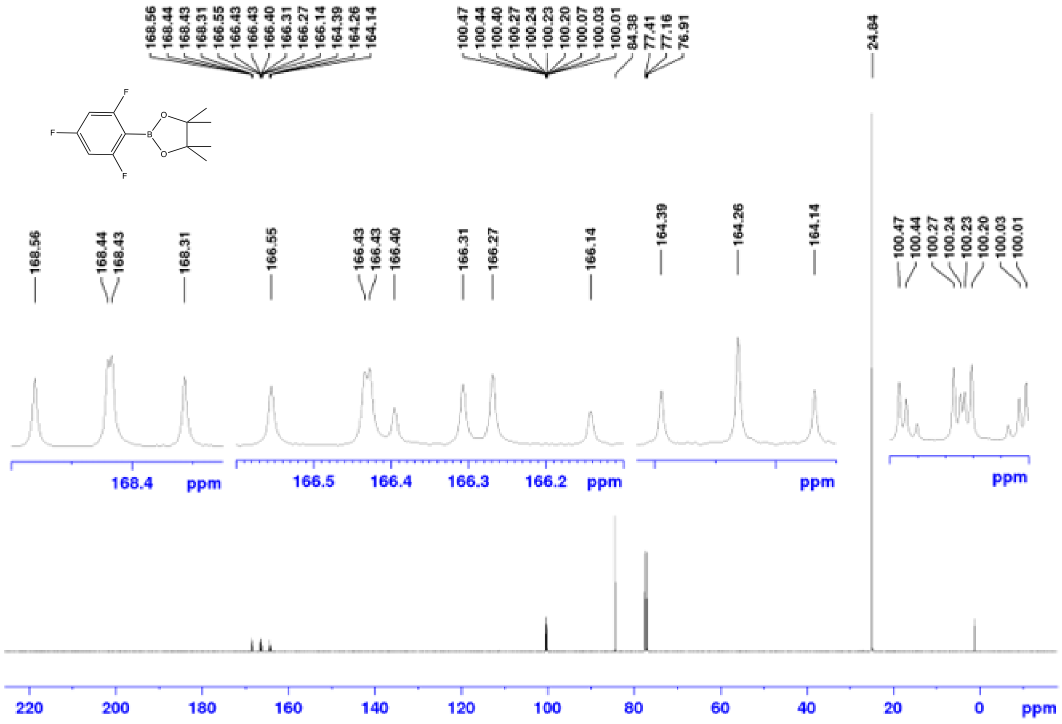

$^{19}F\{^{1}H\}$ NMR spectrum of **2b** ($CDCl_3$, 377 MHz)

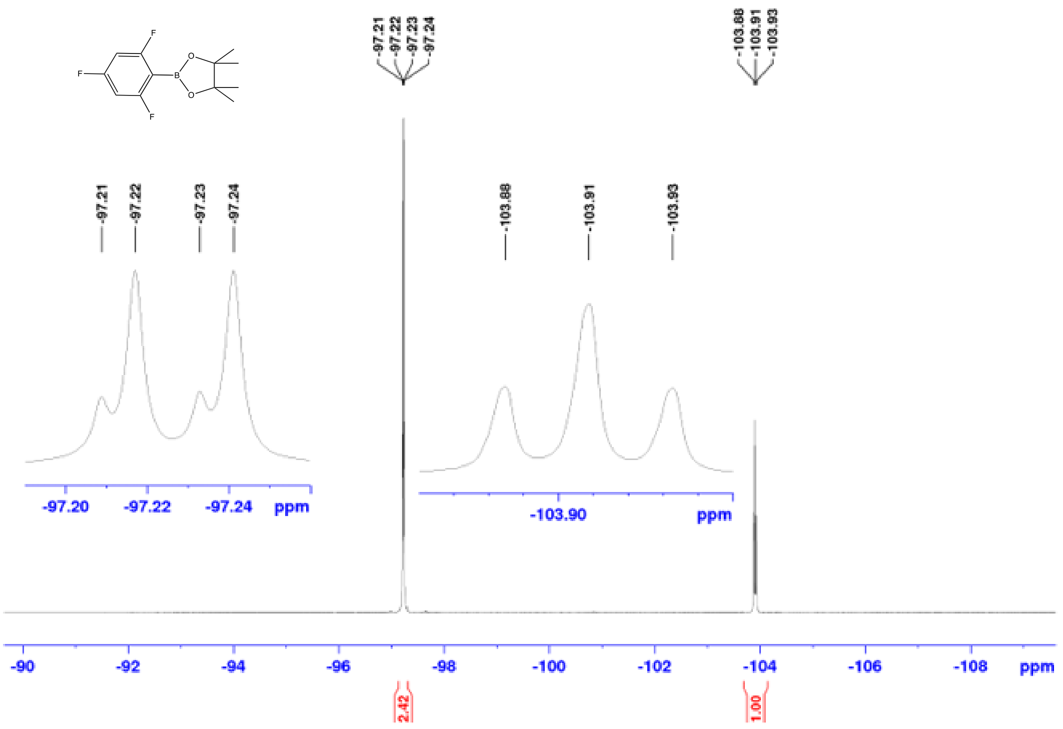

$^{11}B\{^{1}H\}$ NMR spectrum of **2b** (128 MHz, $CDCl_3$)

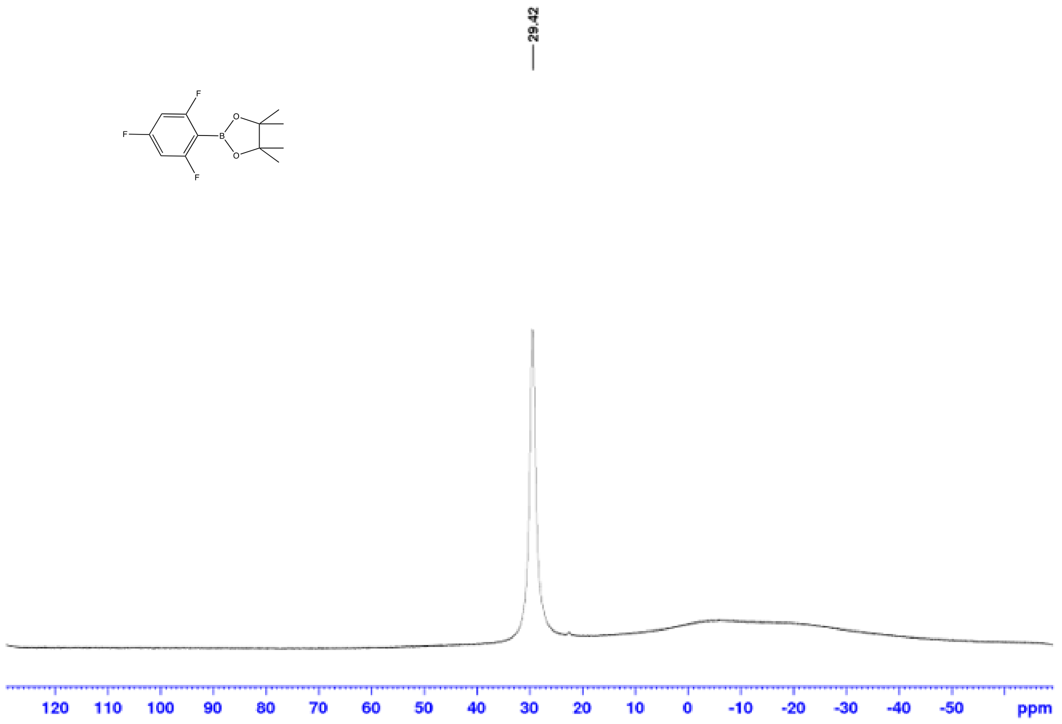

GC-MS of **2b**

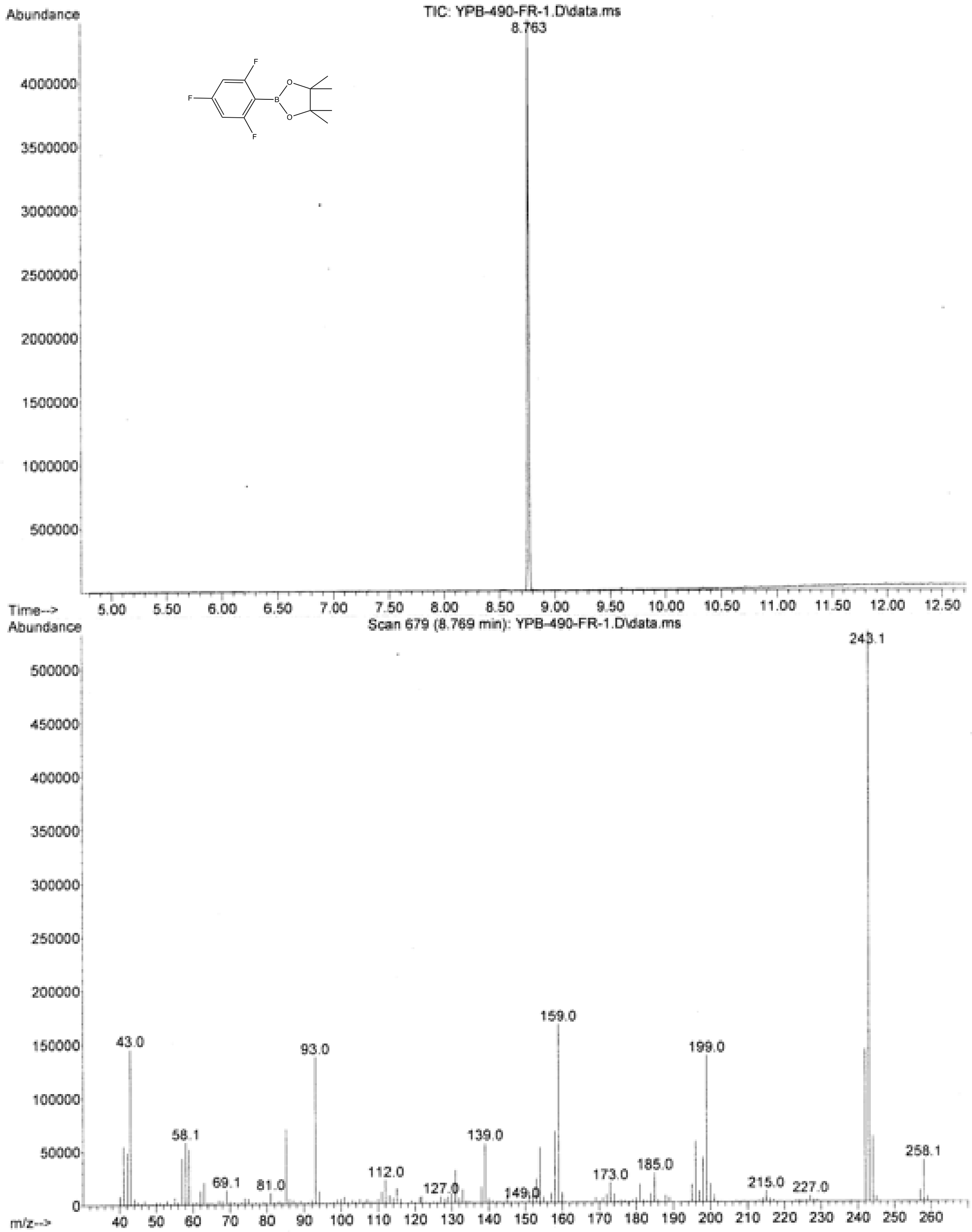
Abundance
TIC: YPB-490-FR-1.D\data.ms
8.763
F
F
F
B
O
O
4000000
3500000
3000000
2500000
2000000
1500000
1000000
500000
Time-->
5.00 5.50 6.00 6.50 7.00 7.50 8.00 8.50 9.00 9.50 10.00 10.50 11.00 11.50 12.00 12.50
Abundance
Scan 679 (8.769 min): YPB-490-FR-1.D\data.ms
243.1
500000
450000
400000
350000
300000
250000
200000
150000
100000
50000
0
159.0
43.0
93.0
199.0
58.1
139.0
258.1
112.0
185.0
173.0
69.1
81.0
127.0
149.0
215.0
227.0
m/z-->
40 50 60 70 80 90 100 110 120 130 140 150 160 170 180 190 200 210 220 230 240 250 260

^{1}H NMR spectrum of **2c** ($CDCl_3$, 400 MHz)

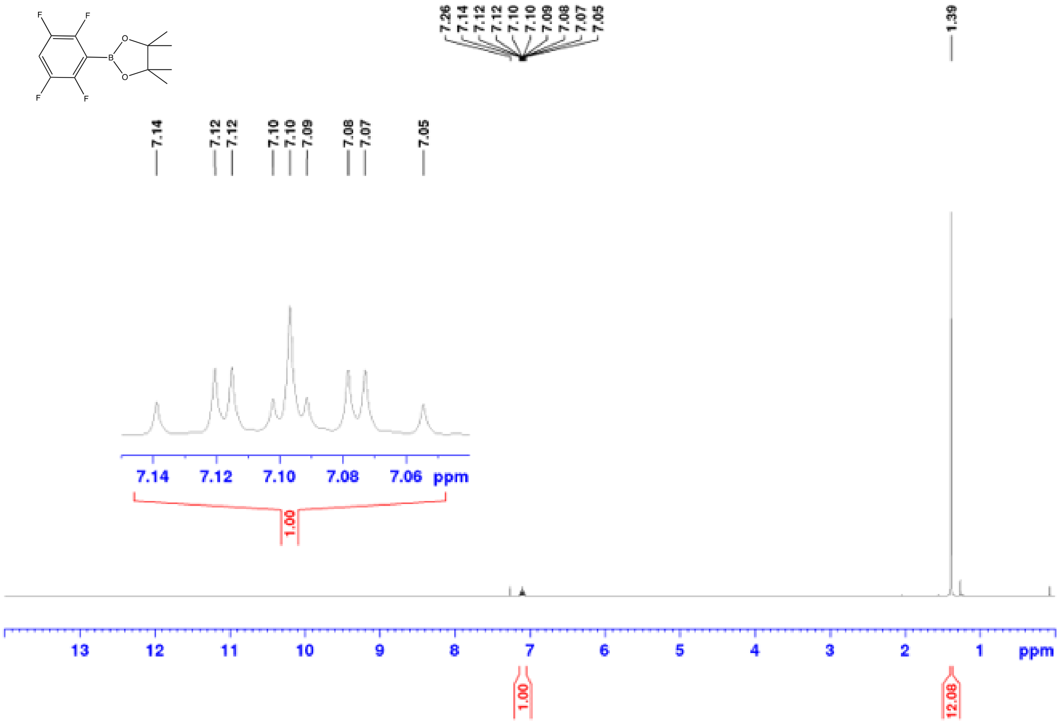

^{13}C{^{1}H} NMR spectrum of **2c** ($CDCl_3$, 126 MHz)

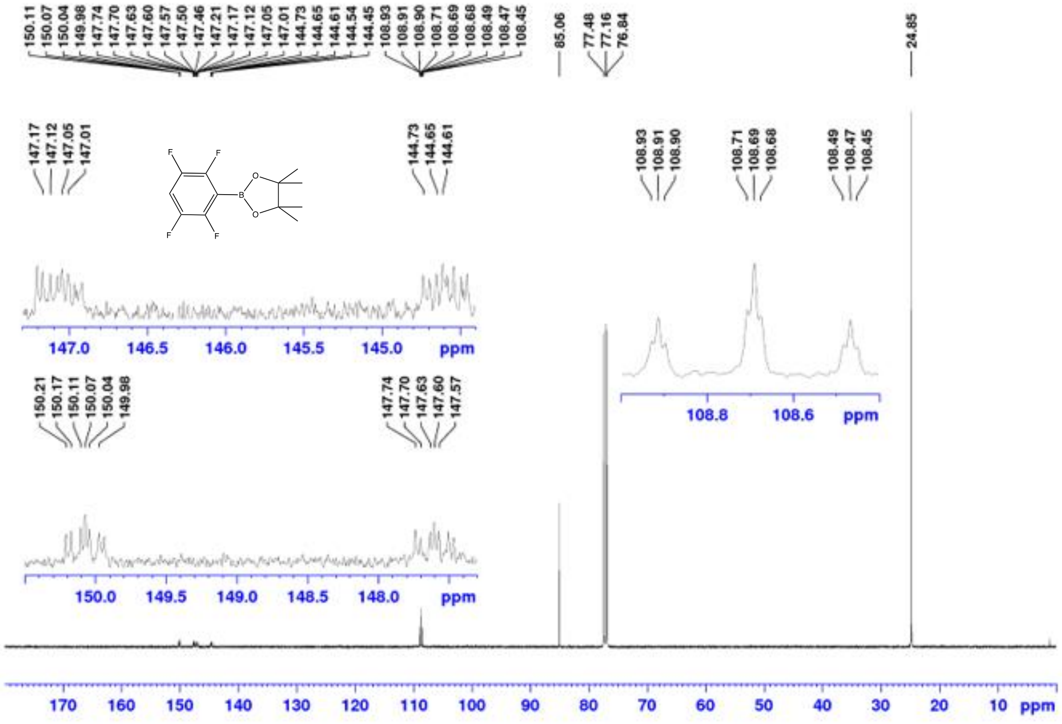

$^{19}F\{^1H\}$ NMR spectrum of **2c** ($CDCl_3$, 377 MHz)

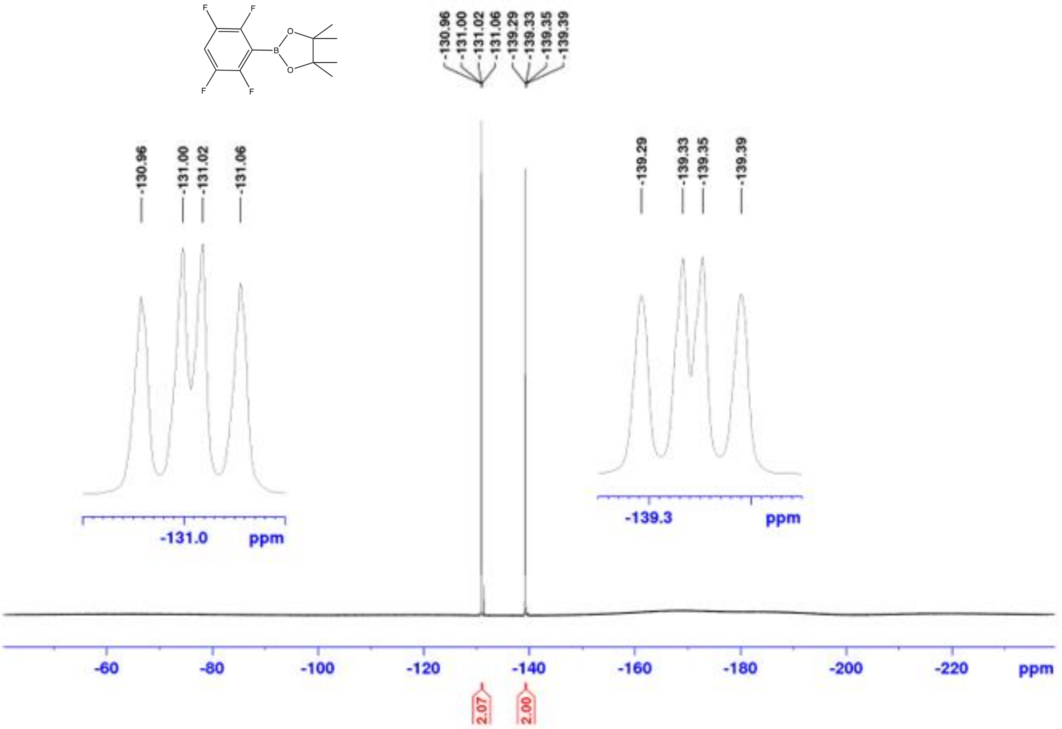

$^{11}B\{^1H\}$ NMR spectrum of **2c** ($CDCl_3$, 128 MHz)

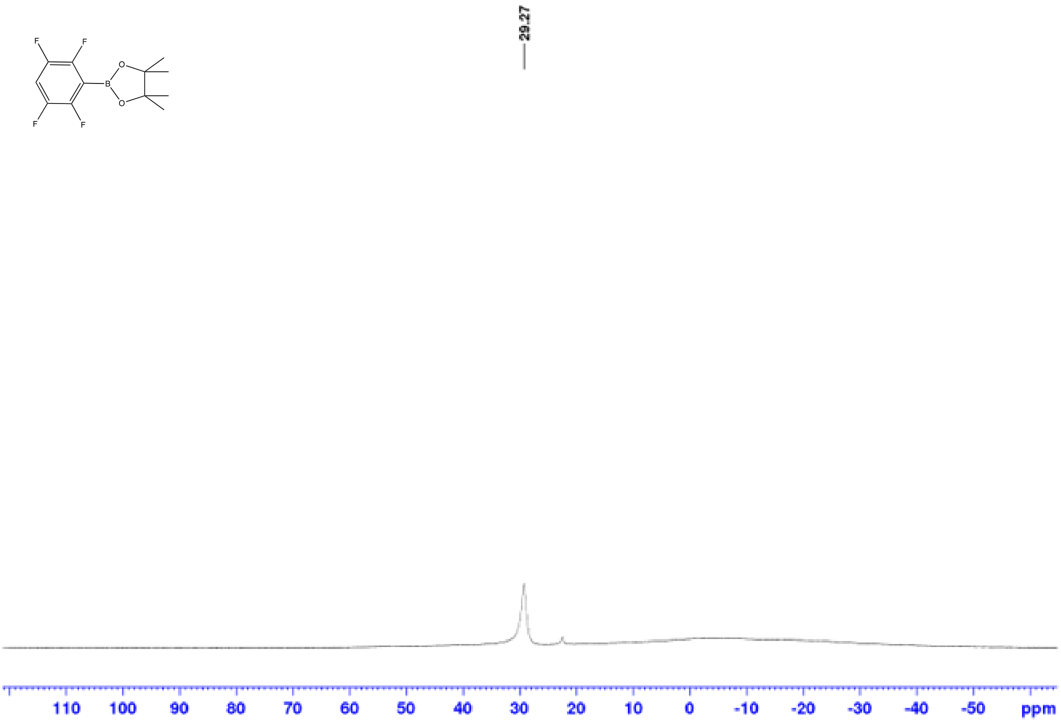

GC-MS of **2c**

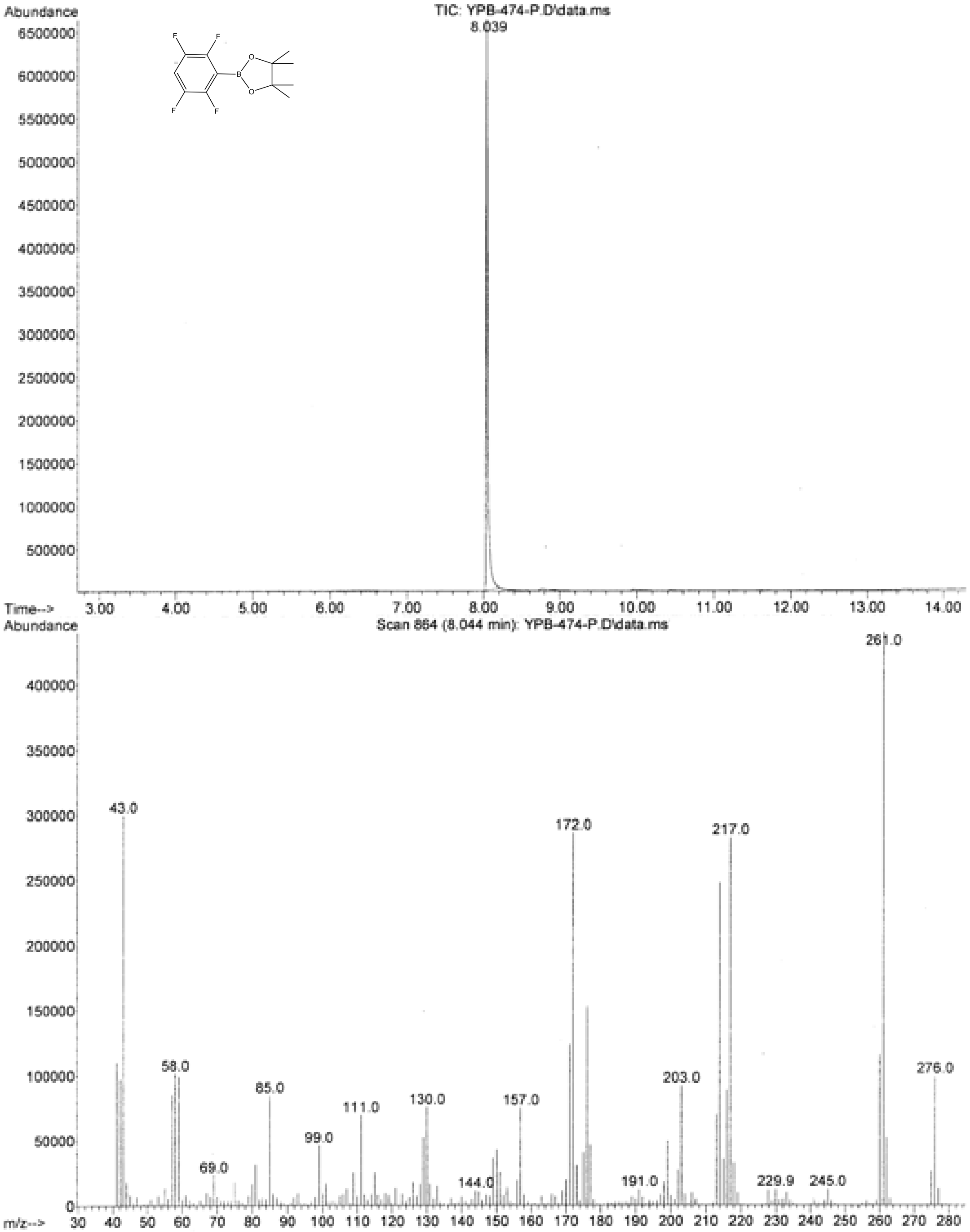
Abundance
TIC: YPB-474-P.D\data.ms
8.039
Time-->
Abundance
Scan 864 (8.044 min): YPB-474-P.D\data.ms
261.0
43.0
172.0
217.0
58.0
85.0
99.0
111.0
130.0
157.0
69.0
144.0
191.0
203.0
229.9
245.0
276.0
m/z-->

^{1}H NMR spectrum of **2d** ($CDCl_3$, 500 MHz)

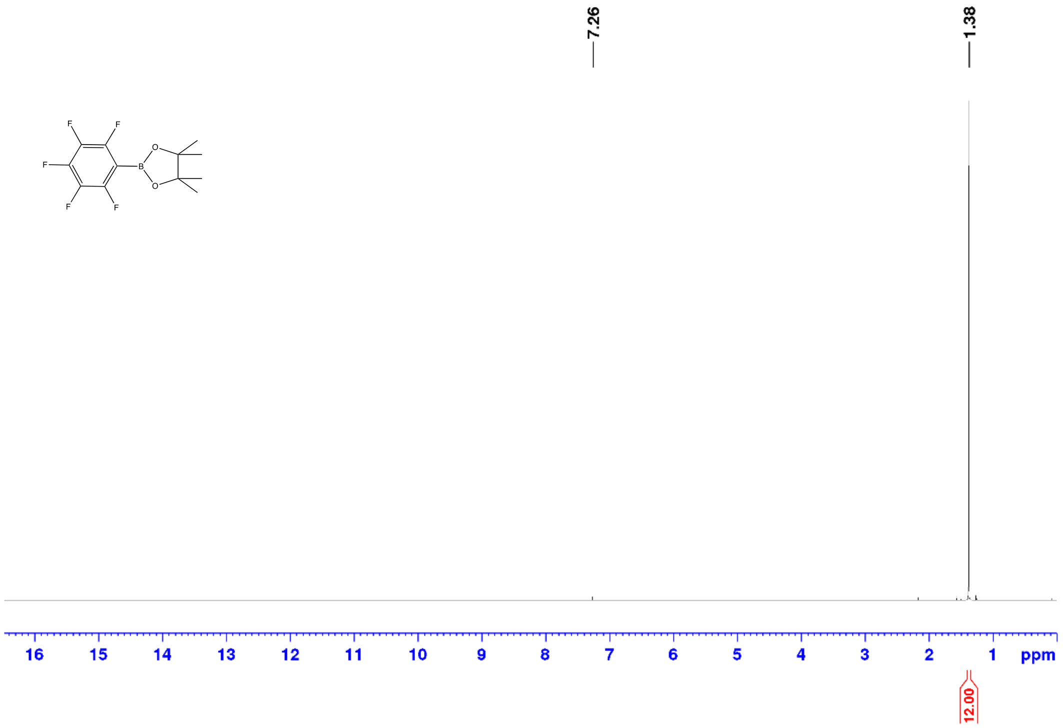

^{13}C{^{1}H} NMR spectrum of **2d** ($CDCl_3$, 126 MHz)

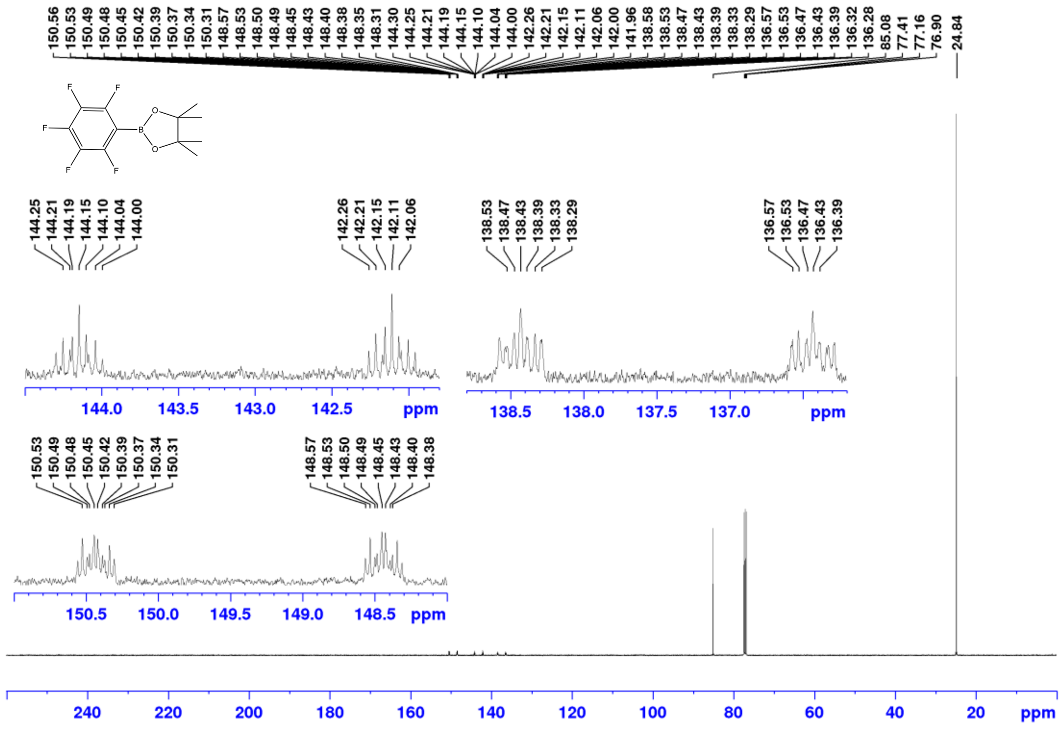

^{19}F NMR spectrum of **2d** ($CDCl_3$, 471 MHz)

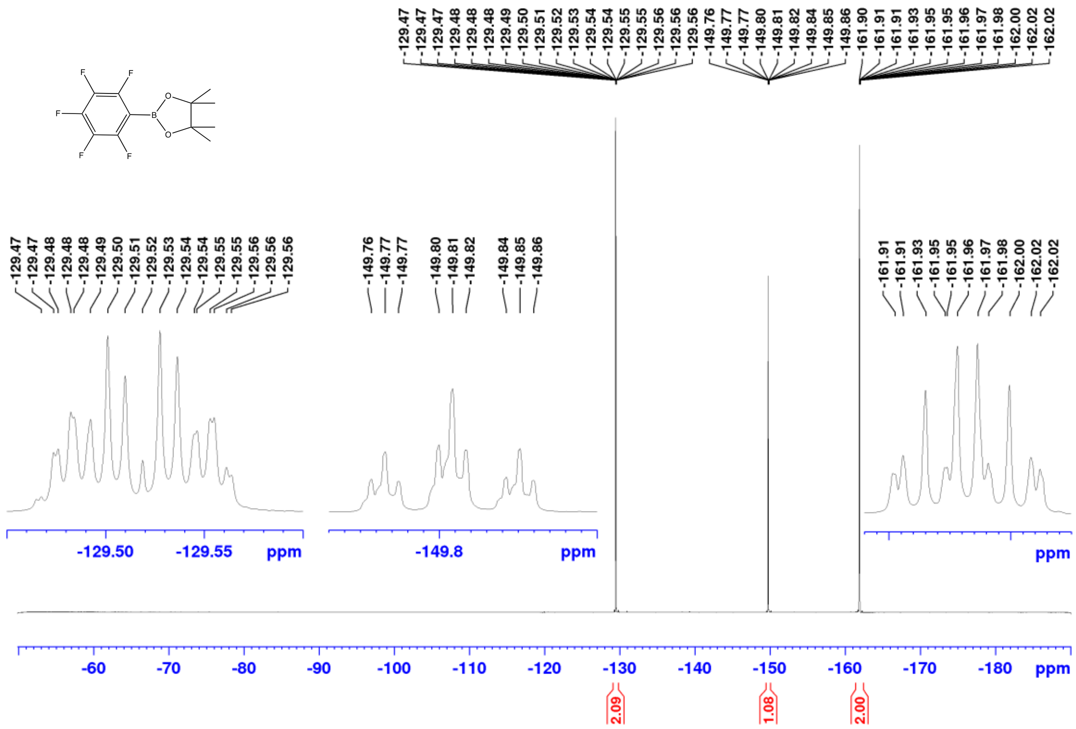

^{11}B{^{1}H} NMR spectrum of **2d** ($CDCl_3$, 96 MHz)

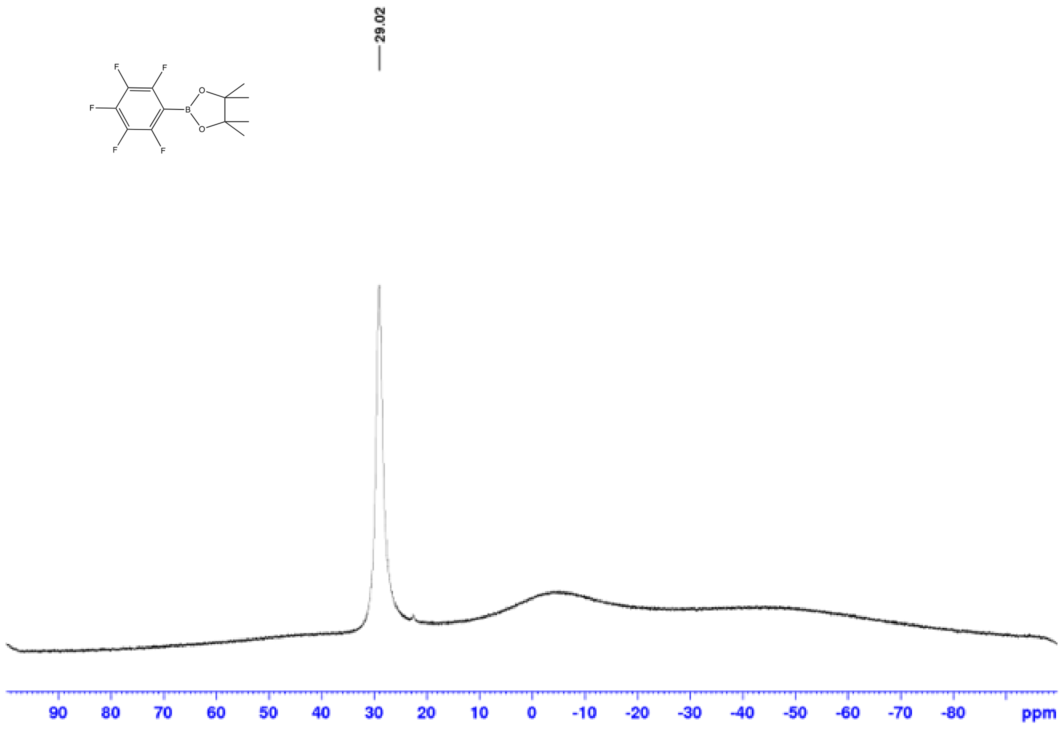

GC-MS of **2d**

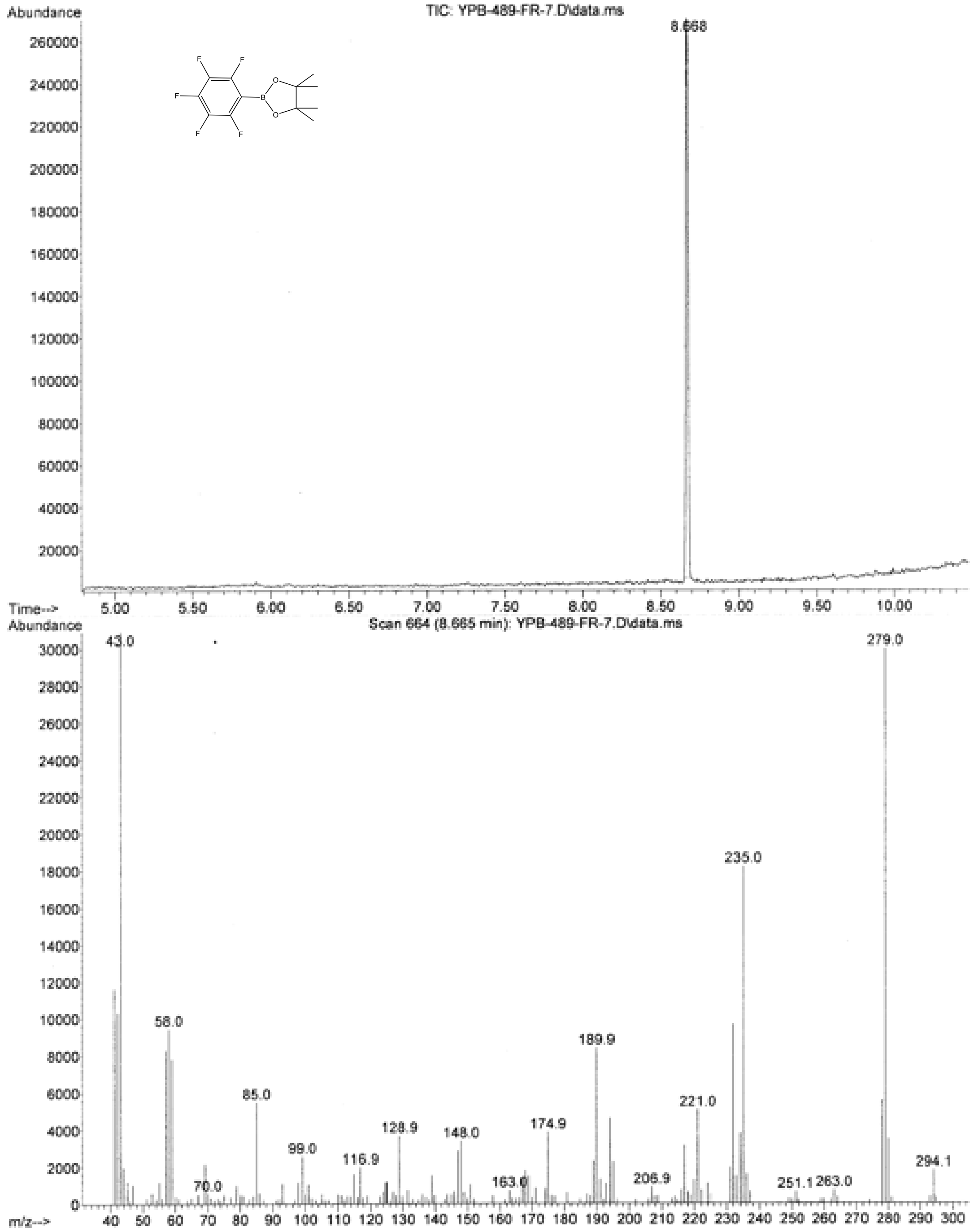

Abundance
TIC: YPB-489-FR-7.D\data.ms
8.668
Time-->
Abundance
Scan 664 (8.665 min): YPB-489-FR-7.D\data.ms
43.0
58.0
85.0
99.0
116.9
128.9
148.0
163.0
174.9
189.9
206.9
221.0
235.0
251.1
263.0
279.0
294.1
m/z-->

^{1}H NMR spectrum of **2e** ($CDCl_3$, 500 MHz)

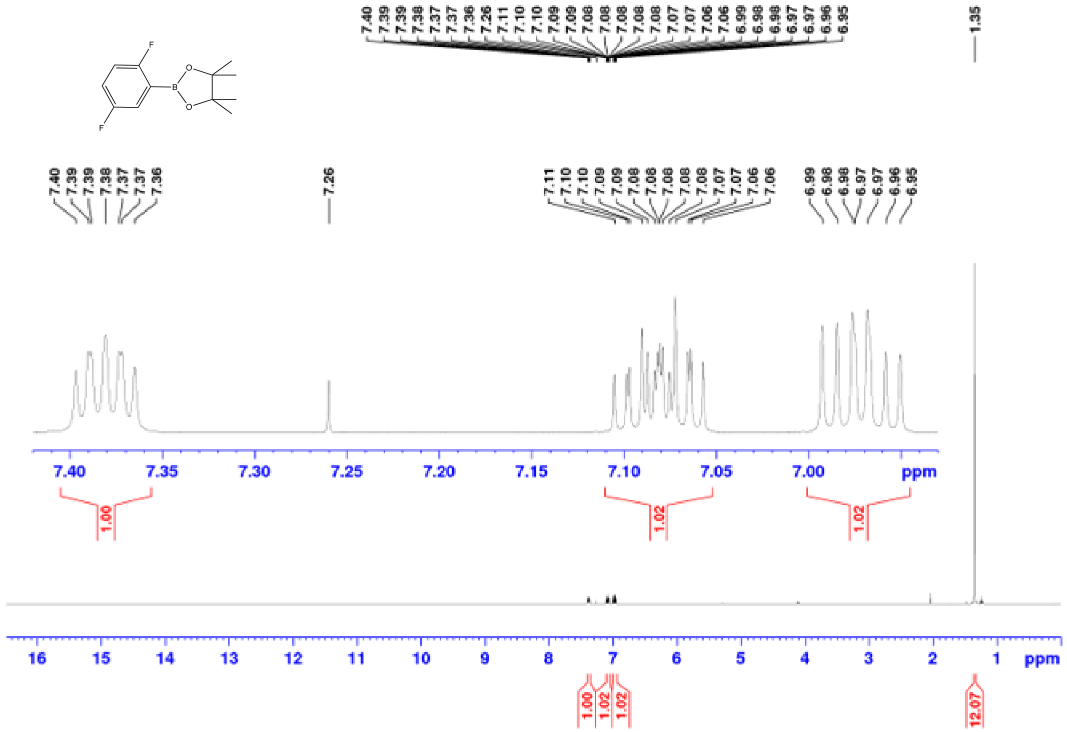

^{13}C{^{1}H} NMR of **2e** ($CDCl_3$, 126 MHz)

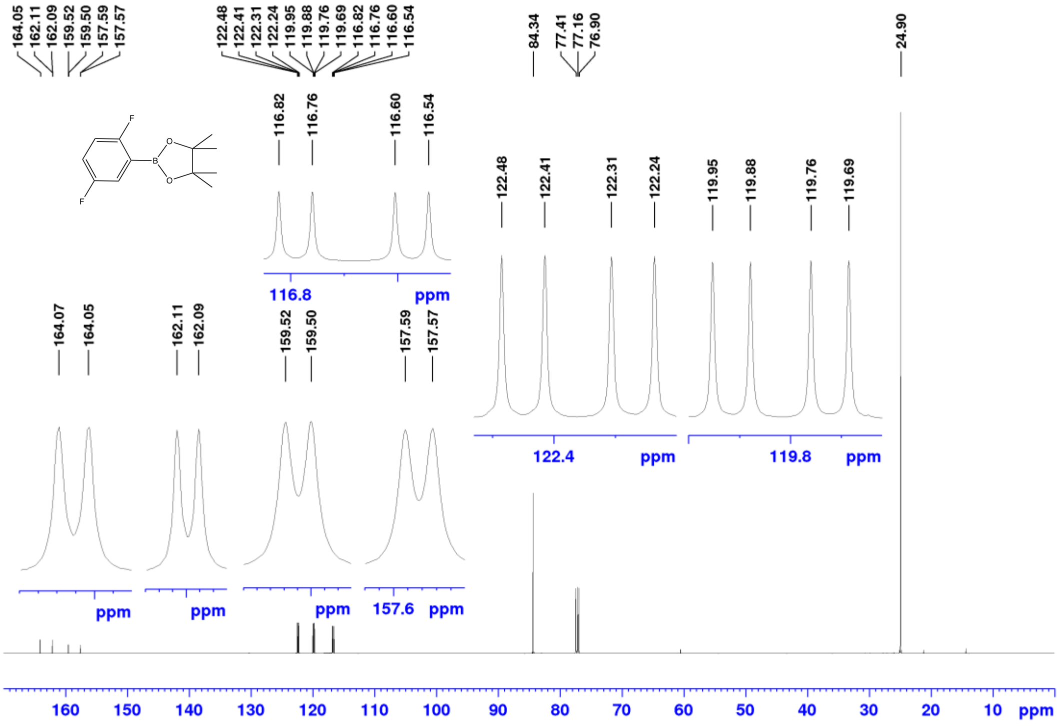

$^{19}F\{^{1}H\}$ NMR **2e** ($CDCl_3$, 377 MHz)

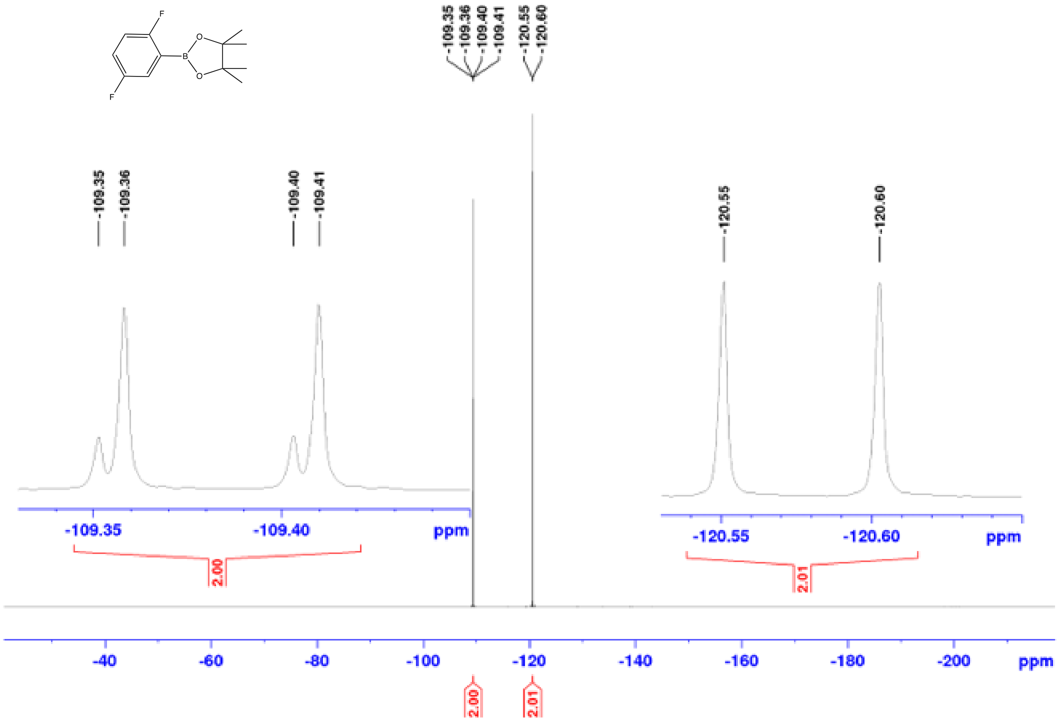

$^{11}B\{^{1}H\}$ NMR spectrum of **2e** ($CDCl_3$ 160 MHz)

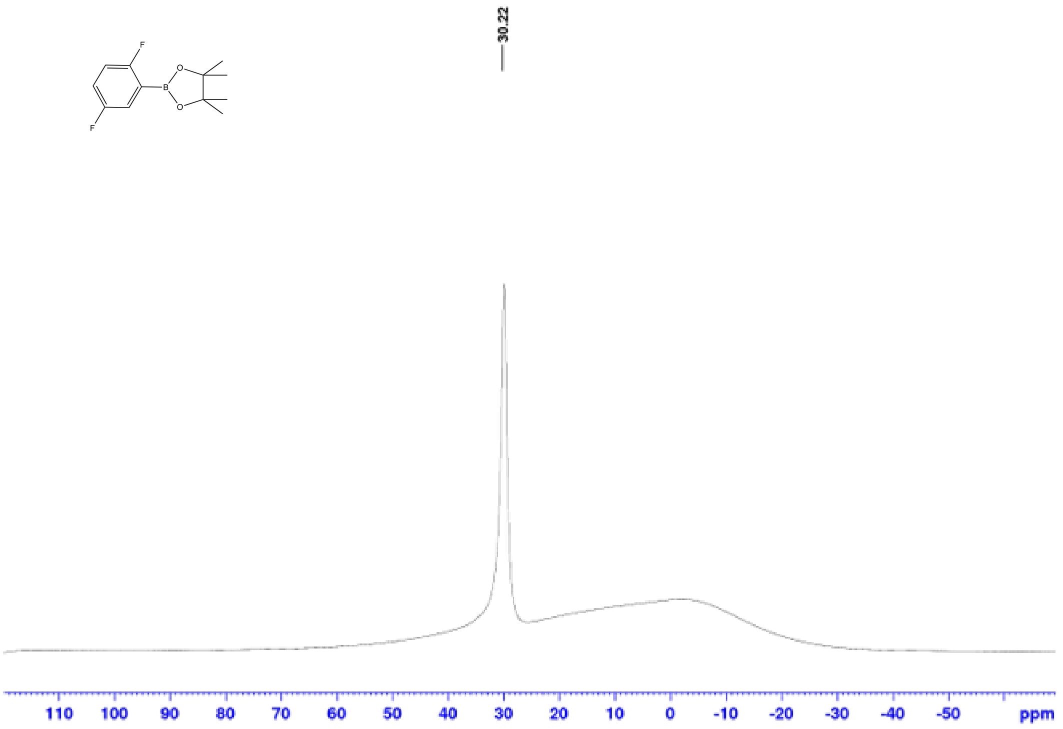

GC-MS of **2e**

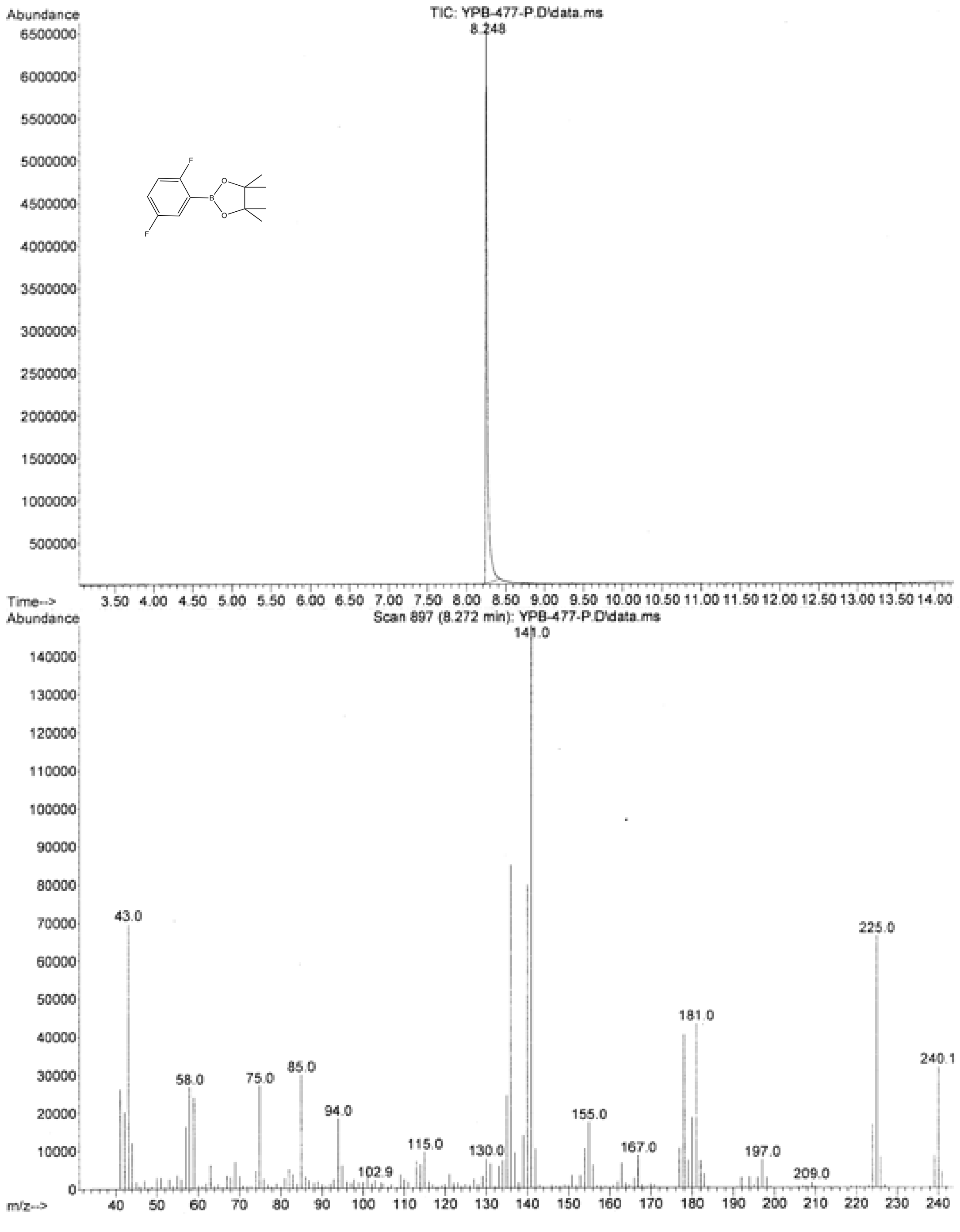

Abundance
TIC: YPB-477-P.D\data.ms
8.248
F
B
O
O
F
Time-->
Abundance
Scan 897 (8.272 min): YPB-477-P.D\data.ms
141.0
43.0
58.0
75.0
85.0
94.0
102.9
115.0
130.0
155.0
167.0
181.0
197.0
209.0
225.0
240.1
m/z-->

^{1}H NMR spectrum of **2f** ($CDCl_3$, 500 MHz)

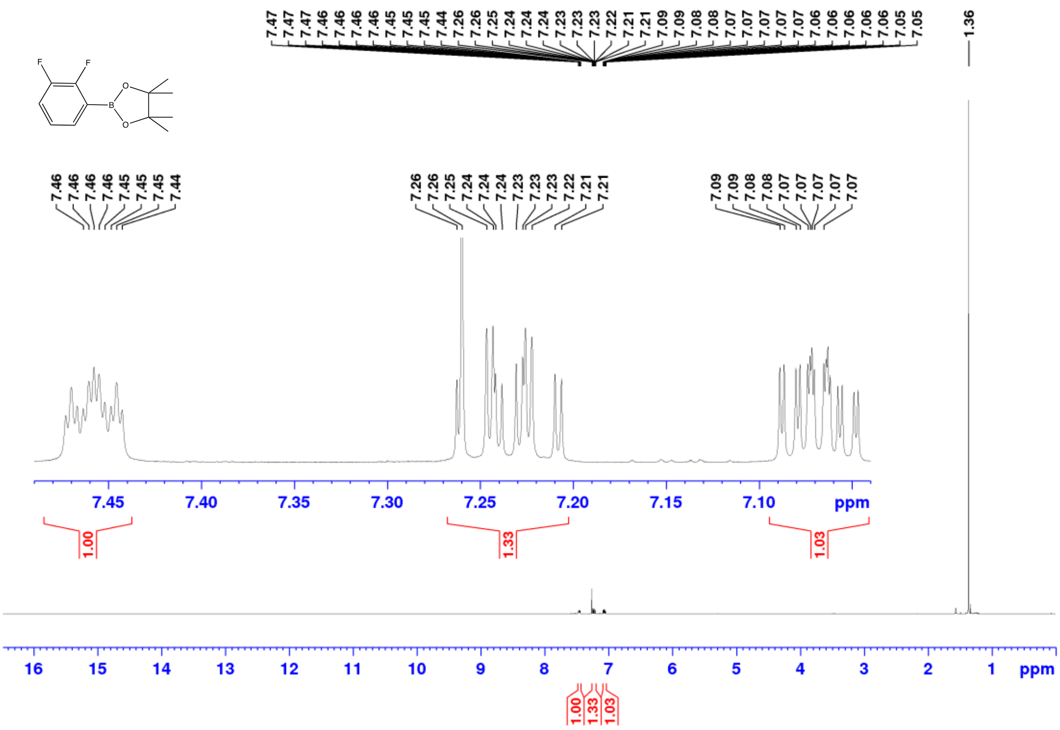

^{13}C{^{1}H} NMR of **2f** ($CDCl_3$, 126 MHz)

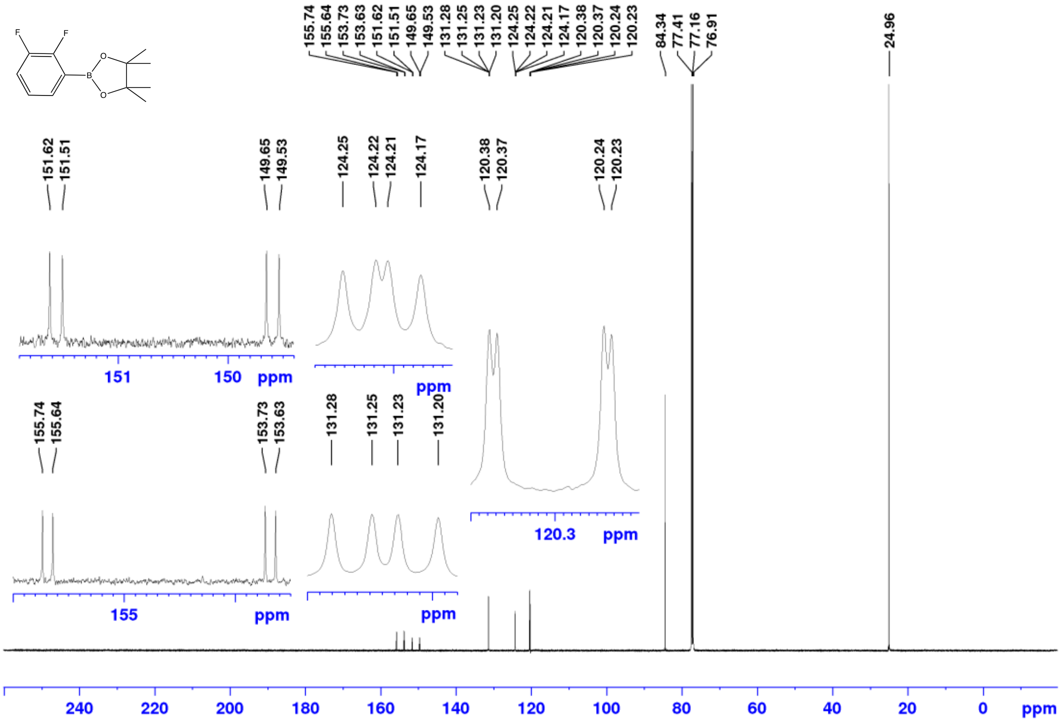

$^{19}F\{^{1}H\}$ NMR **2f** ($CDCl_3$, 377 MHz)

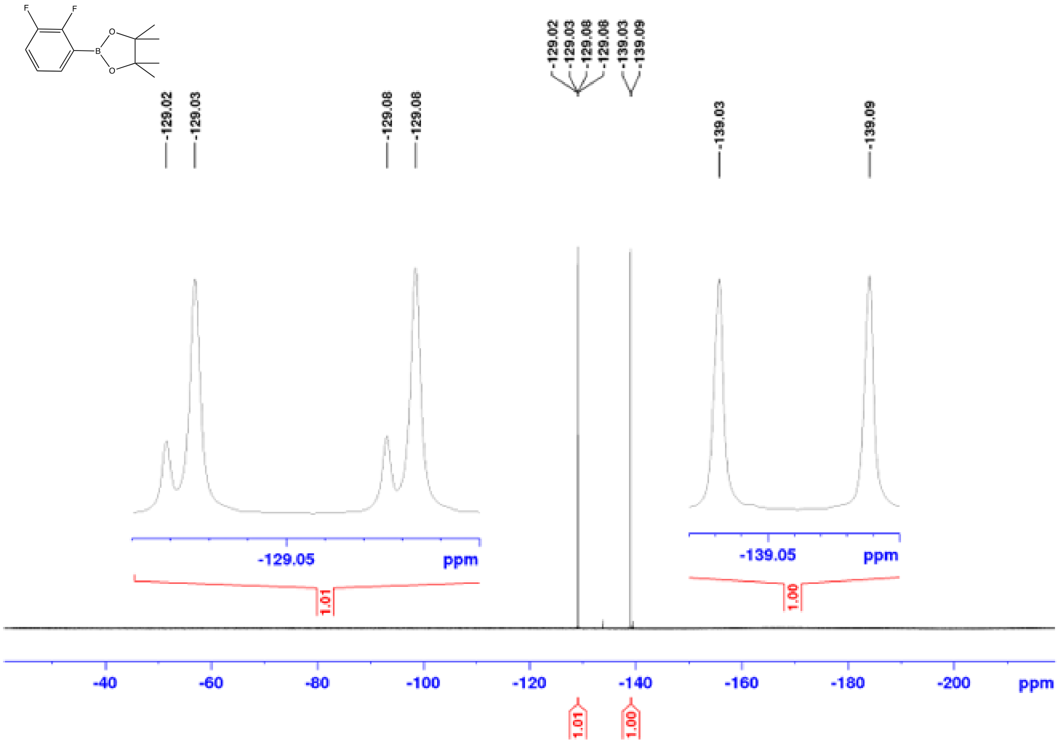

$^{11}B\{^{1}H\}$ NMR spectrum of **2f** ($CDCl_3$ 160 MHz)

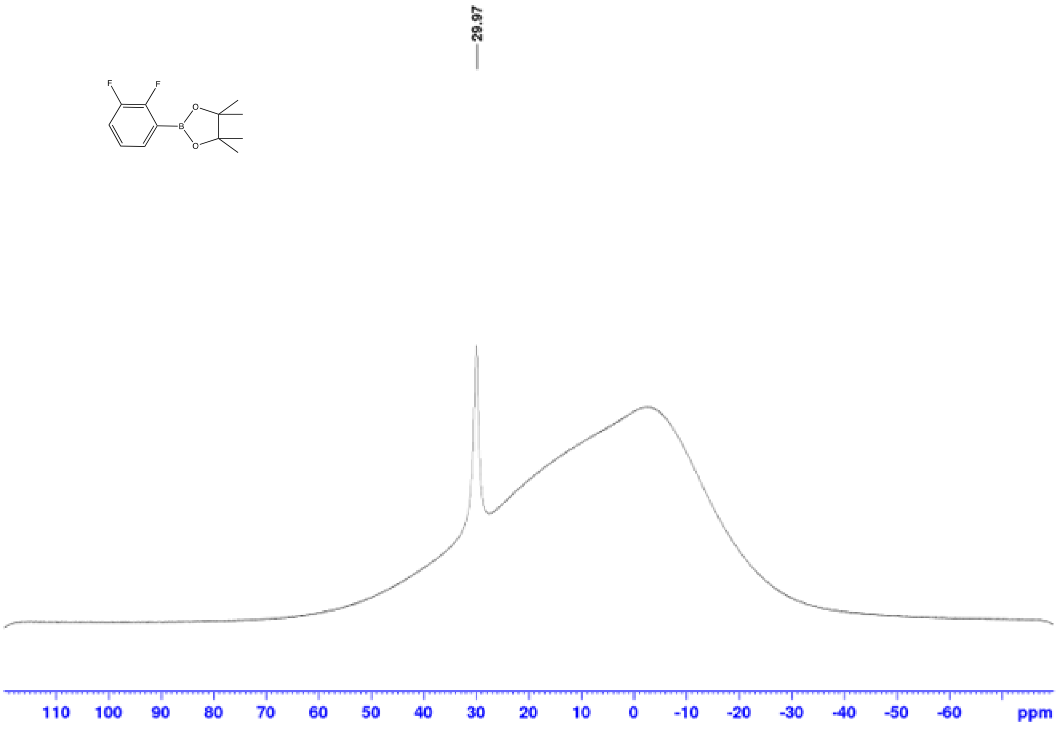

GC-MS of **2f**

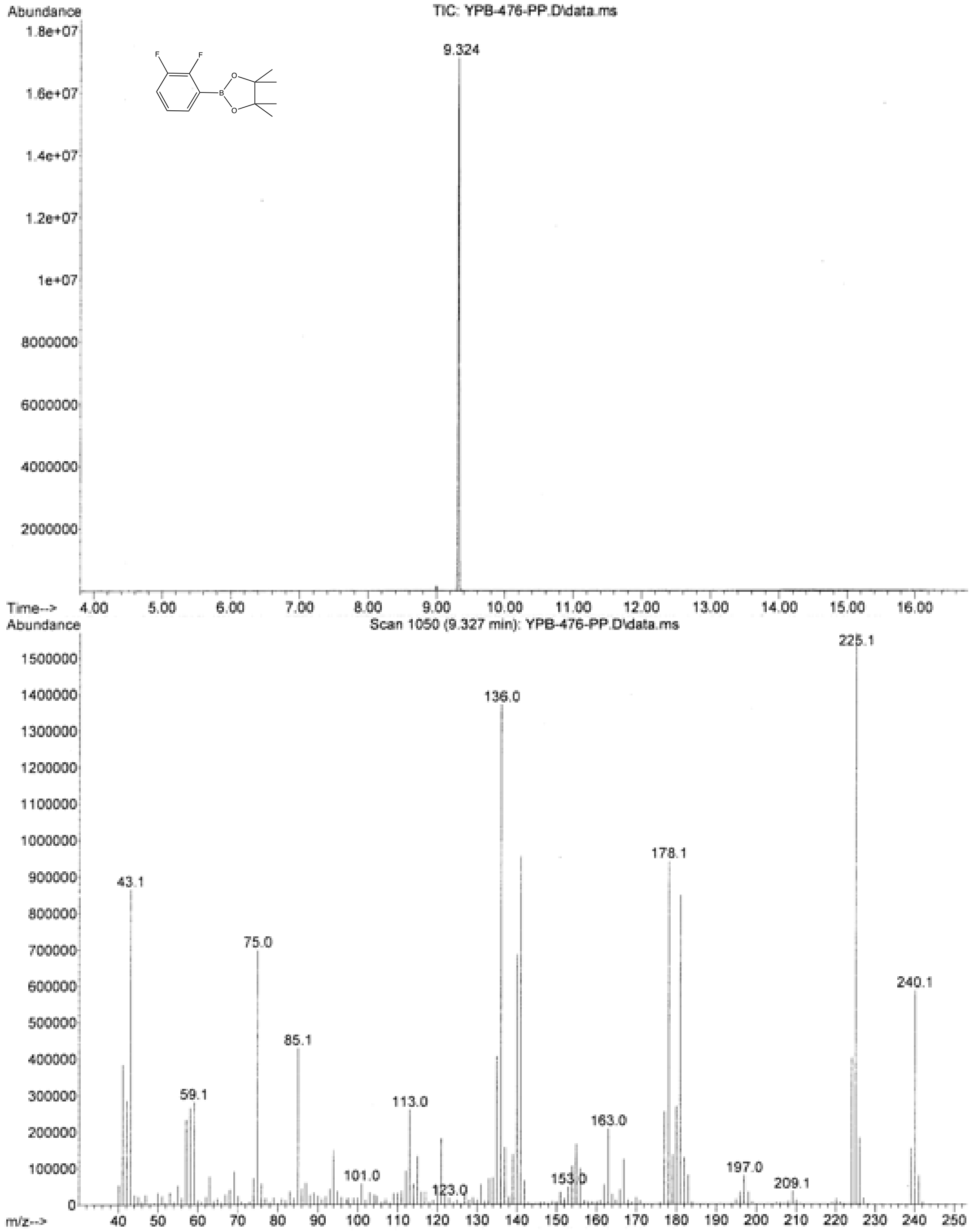
Abundance
TIC: YPB-476-PP.D\data.ms
9.324
F
F
B
O
O
Time-->
Abundance
Scan 1050 (9.327 min): YPB-476-PP.D\data.ms
225.1
136.0
178.1
43.1
75.0
240.1
85.1
59.1
113.0
163.0
197.0
101.0
153.0
209.1
123.0
m/z-->

^{1}H NMR spectrum of **2g** (CDCl3, 500 MHz)

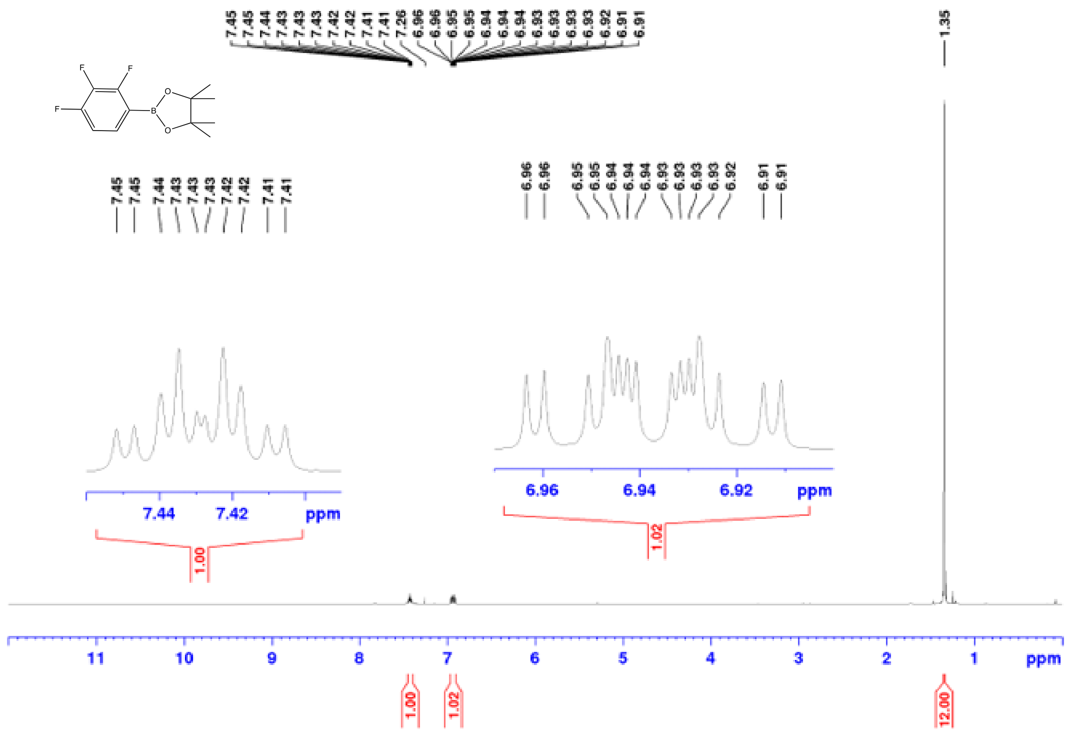

^{13}C{^{1}H} NMR of **2g** ($CDCl_3$, 126 MHz)

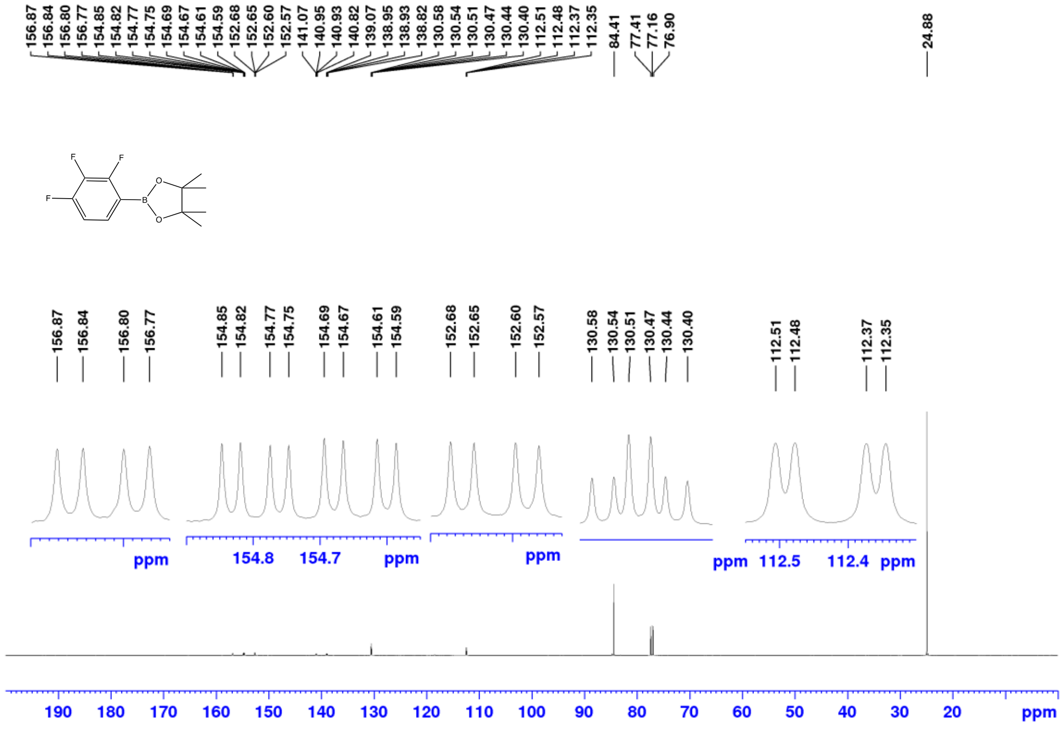

$^{19}F\{^1H\}$ NMR of **2g** ($CDCl_3$, 377 MHz)

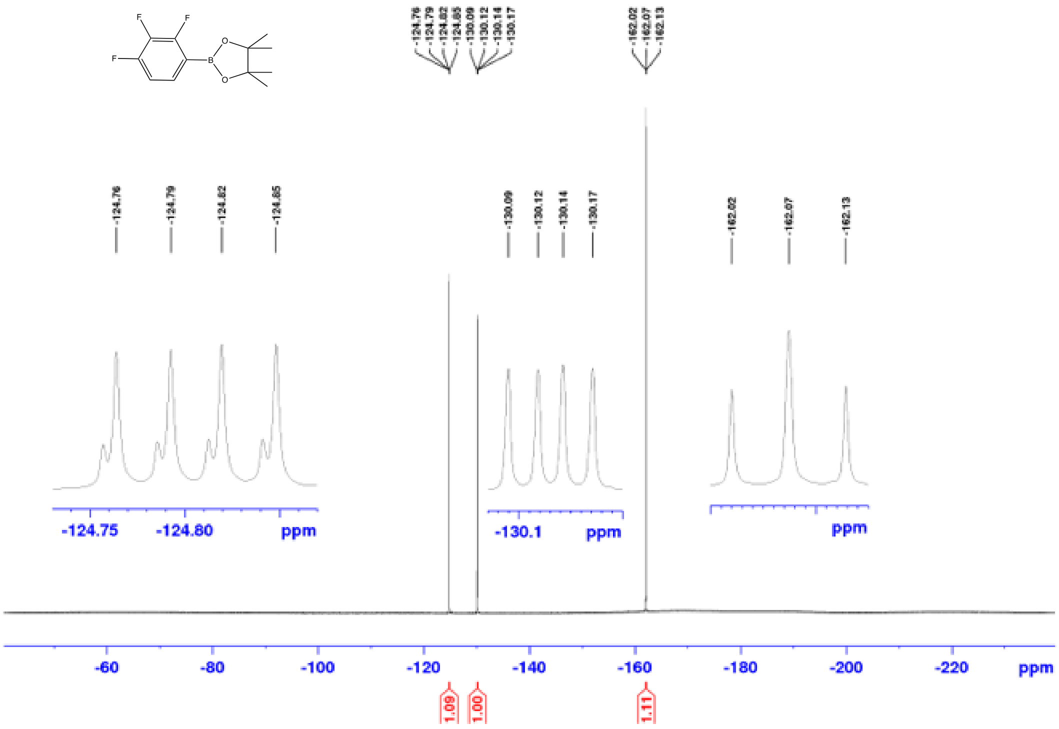

$^{11}B\{^1H\}$ NMR of **2g** ($CDCl_3$, 128 MHz)

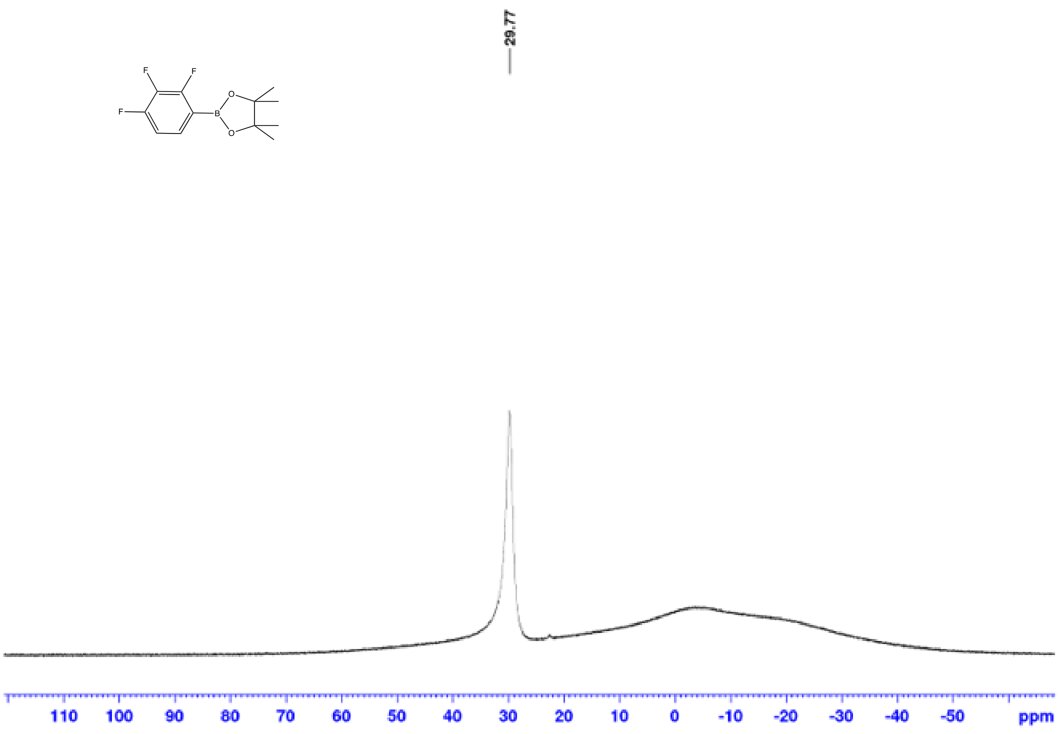

GC-MS of **2g**

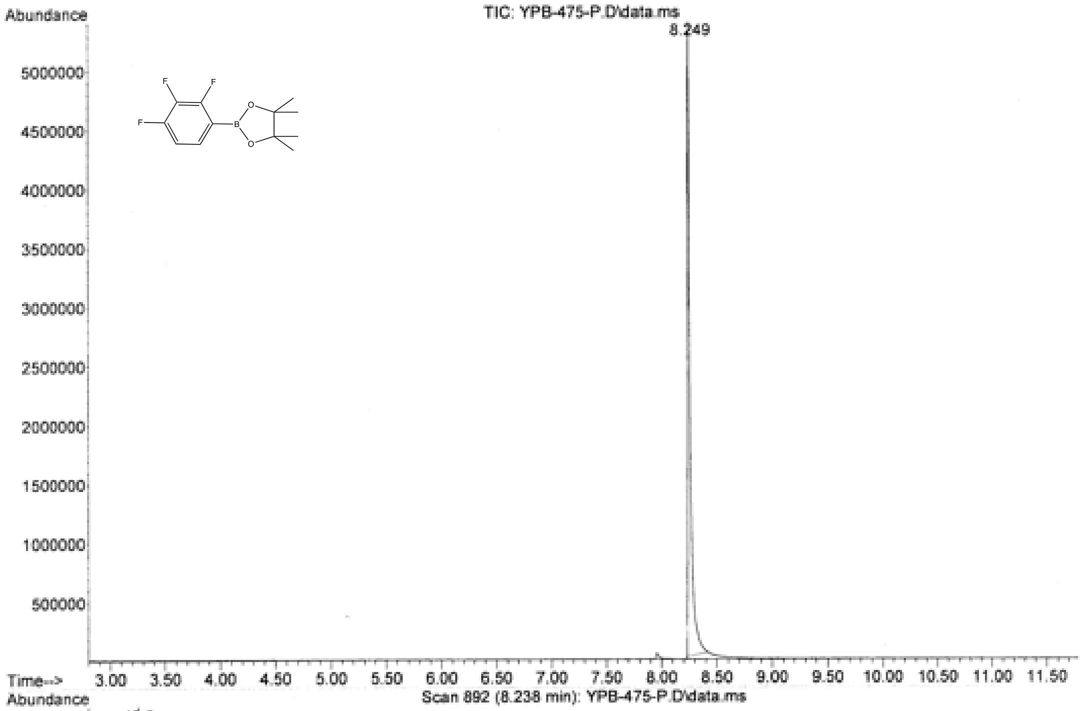
Abundance
TIC: YPB-475-P.D\data.ms
8.249
Time-->
F
F
F
B
O
O

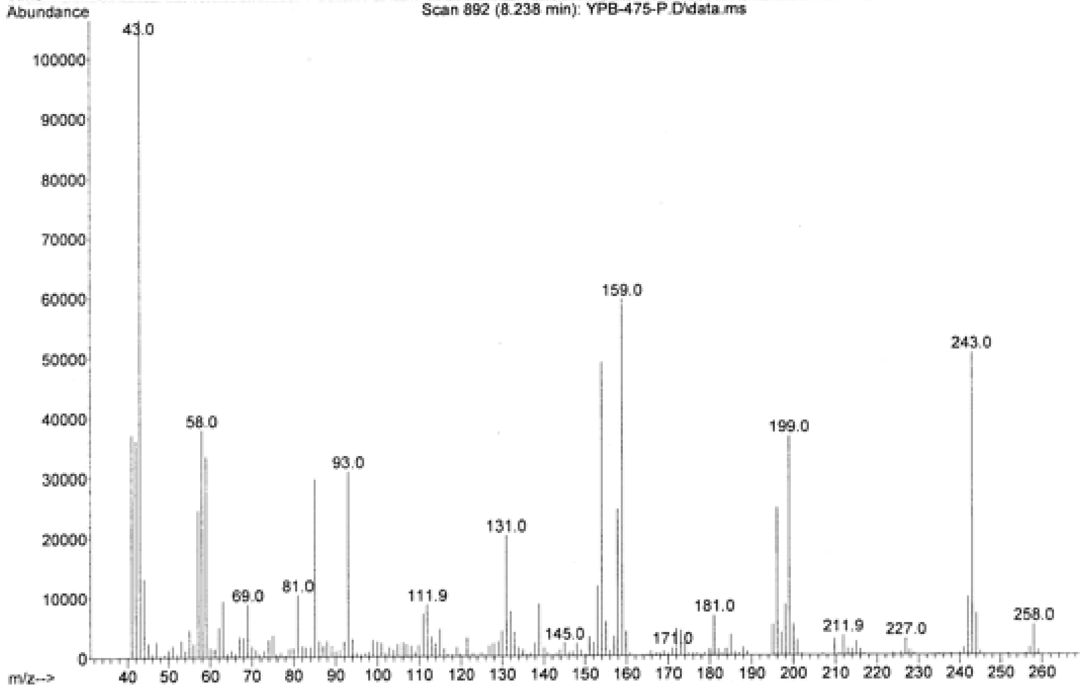
Abundance
Scan 892 (8.238 min): YPB-475-P.D\data.ms
43.0
58.0
69.0
81.0
93.0
111.9
131.0
145.0
159.0
171.0
181.0
199.0
211.9
227.0
243.0
258.0
m/z-->

^{1}H NMR spectrum of **2h** ($CDCl_3$, 500 MHz)

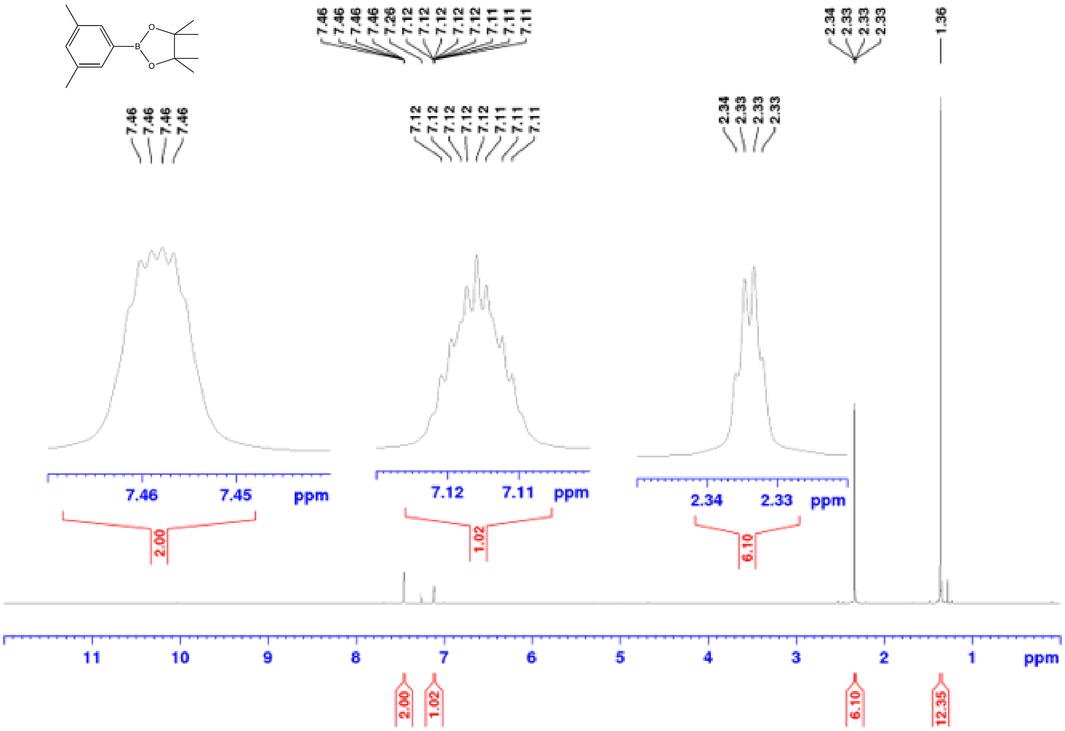

$^{13}C\{^{1}H\}$ NMR of **2h** ($CDCl_3$, 75 MHz)

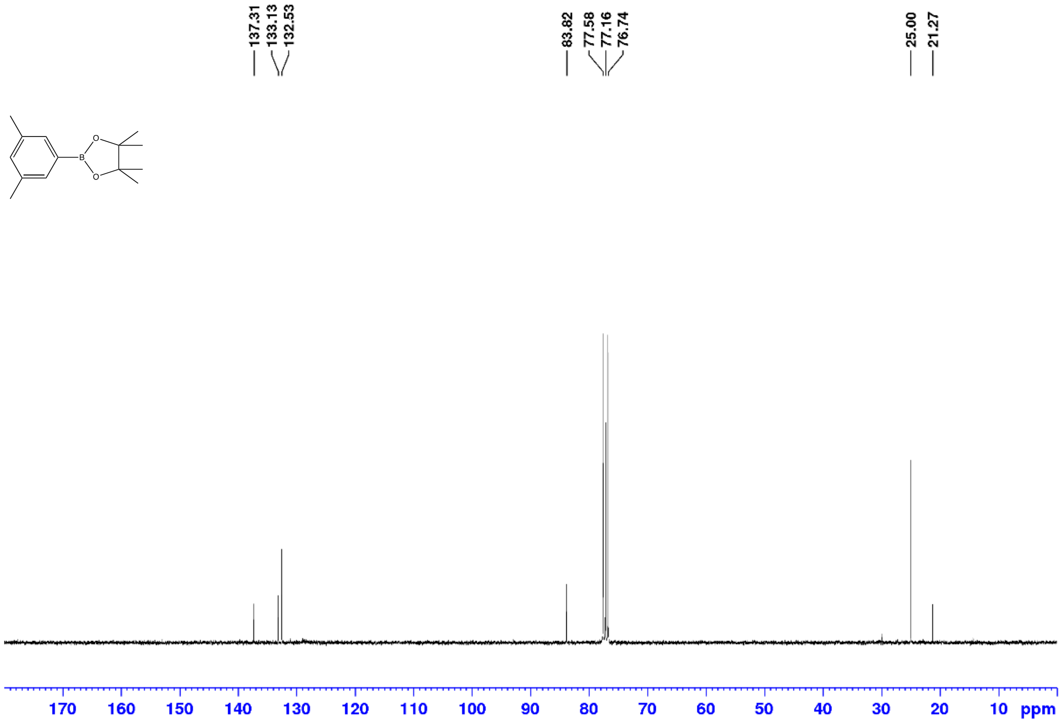

$^{11}B\{^{1}H\}$ NMR of **2h** (96 MHz, $CDCl_3$)

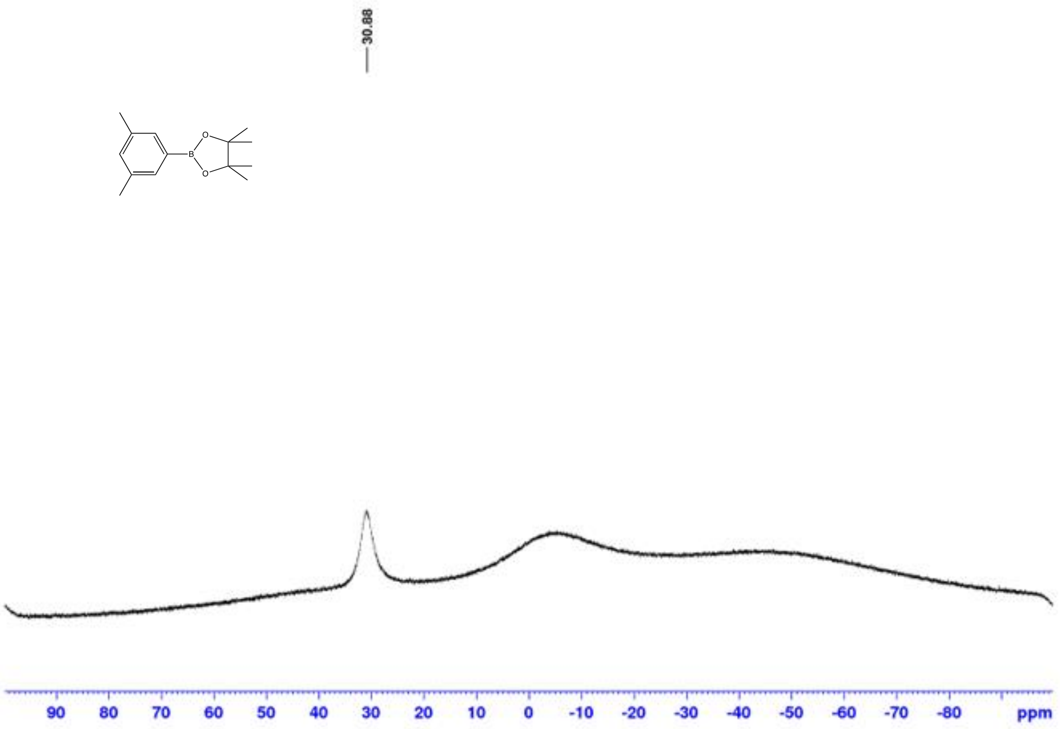

GC-MS of **2h**

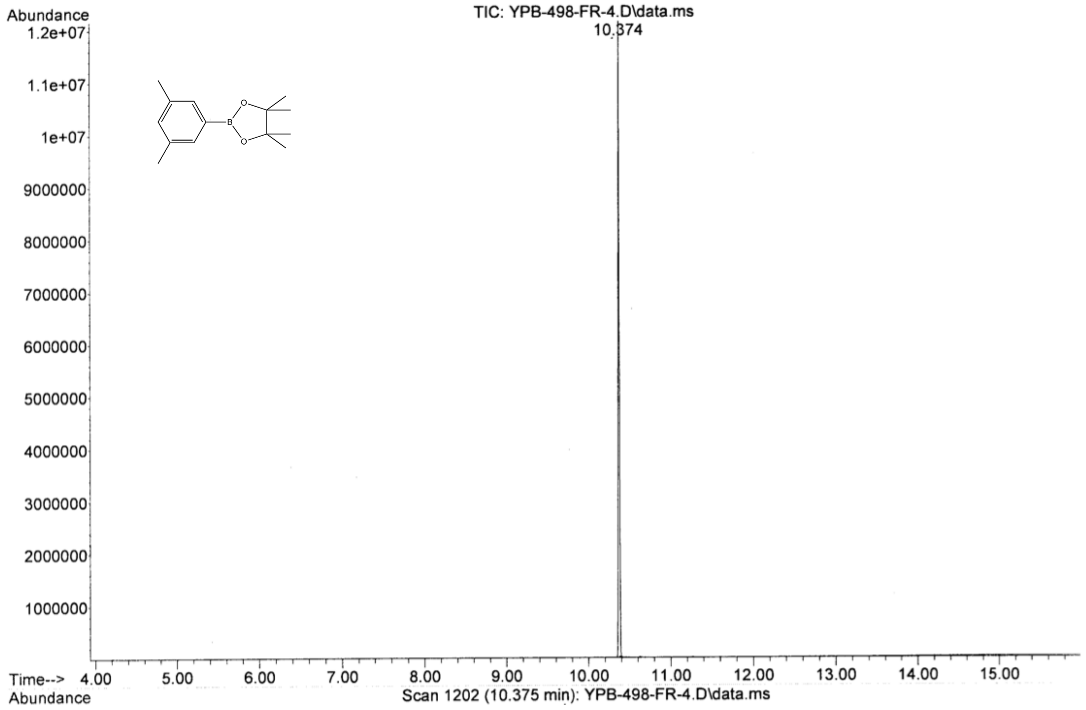

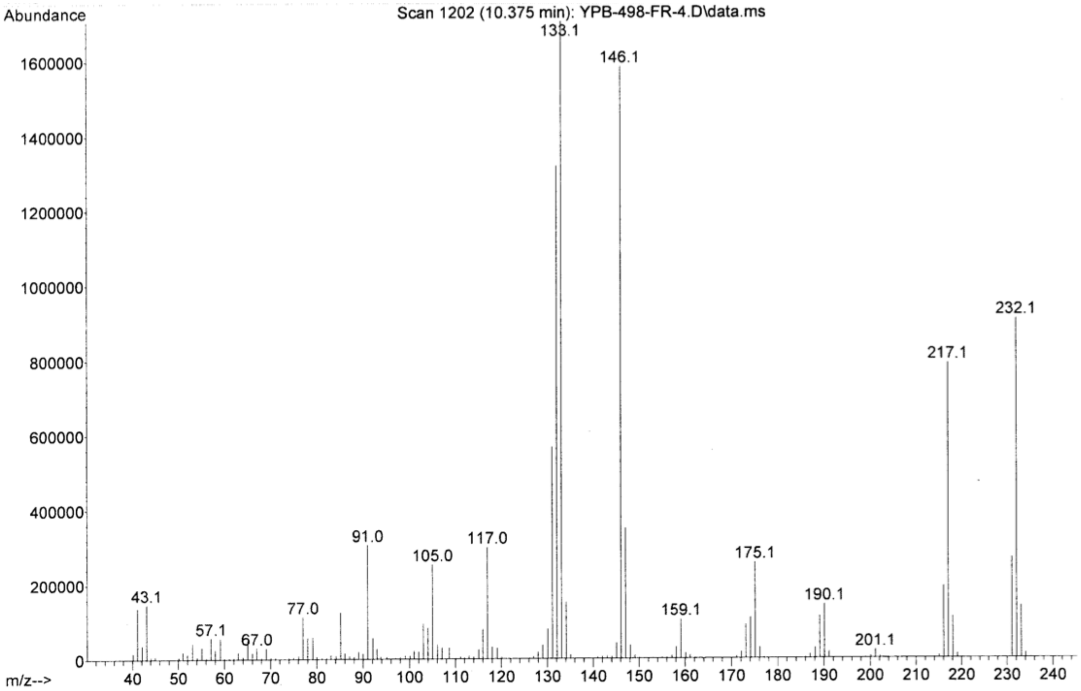
Abundance
Scan 1202 (10.375 min): YPB-498-FR-4.D\data.ms
133.1
146.1
232.1
217.1
91.0
105.0
117.0
175.1
43.1
159.1
190.1
57.1
77.0
67.0
201.1
m/z-->

References

[S1] J. Takaya, S. Ito, H. Nomomoto, N. Saito, N. Kirai, N. Iwasawa, *Chem. Commun*. **2015**, *51*, 17662–17665.

[S2] G. A. Chotana, M. A. Rak and M. R. Smith, *J. Am. Chem. Soc.* **2005**, 127, 10539–10544.

[S3] T. Furukawa, M. Tobisu, N. Chatani, *J. Am. Chem. Soc.* **2015**, *137*, 12211–12214.

[S4] H. Ren, Y.-P. Zhou, Y. Bai, C. Cui, M. Driess, *Chem. Eur. J.* **2017**, *23*, 5663–5667.

[S5] P. Harrisson, J. Morris, T. B. Marder, P. Steel, *Org. Lett.* **2009**, *11*, 3586–3589.
